# An Integrated Approach of QTL Mapping and Genome-Wide Association Analysis Identifies Candidate Genes for Phytophthora Blight Resistance in Sesame (*Sesamum indicum* L.)

**DOI:** 10.3389/fpls.2021.604709

**Published:** 2021-02-16

**Authors:** Sovetgul Asekova, Eunyoung Oh, Krishnanand P. Kulkarni, Muhammad Irfan Siddique, Myoung Hee Lee, Jung In Kim, Jeong-Dong Lee, Minsu Kim, Ki-Won Oh, Tae-Joung Ha, Sung-Up Kim, Kwang-Soo Cho

**Affiliations:** ^1^Department of Southern Area Crop Science, National Institute of Crop Science, RDA, Miryang-si, South Korea; ^2^School of Applied Biosciences, Kyungpook National University, Daegu, South Korea; ^3^Department of Plant Science, Plant Genomics and Breeding Institute, College of Agriculture and Life Sciences, Seoul National University, Seoul, South Korea

**Keywords:** Sesame, Phytophthora blight, QTL, GBS, GWAS, qRT-PCR

## Abstract

Phytophthora blight (PB) caused by *Phytophthora nicotianae* is a highly destructive disease in sesame (*Sesamum indicum* L.). In this study, we used linkage mapping and genome-wide association study (GWAS) to identify quantitative trait loci (QTL) and candidate genes associated with PB resistance. The QTL mapping in 90 RILs of the Goenbaek × Osan cross using genotyping-by-sequencing detected significant QTLs for PB resistance on chromosome 10, explaining 12.79%–13.34% of phenotypic variation. Association of this locus to PB resistance was also revealed through bulked segregant analysis in second RIL population (Goenbaek × Milsung cross) comprising 188 RILs. The GWAS of 87 sesame accessions evaluated against three *P. nicotianae* isolates identified 29 SNPs on chromosome 10 significantly associated with PB resistance. These SNPs were located within a 0.79 Mb region, which co-located with the QTL intervals identified in RIL populations, and hence scanned for identifying candidate genes. This region contained several defense-related candidate *R* genes, five of which were selected for quantitative expression analysis. One of these genes, *SIN_1019016* was found to show significantly higher expression in the resistant parent compared to that in the susceptible parents and selected RILs. Paired-end sequencing of the gene *SIN_1019016* in parental cultivars revealed two synonymous SNPs between Goenbaek and Osan in exon 2 of coding DNA sequence. These results suggested *SIN_1019016* as one of the candidate gene conferring PB resistance in sesame. The findings from this study will be useful in the marker-assisted selection as well as the functional analysis of PB resistance candidate gene(s) in sesame.

## Introduction

Sesame (*Sesamum indicum* L.) (2*n* = 2*x* = 26), with a genome of 357 Mb arranged in 13 chromosomes, is an orphan crop with limited genomic resources (Wang et al., 2016). Among edible oilseed crops, sesame occupies a unique position—it can be cultivated throughout the year and its polyunsaturated fatty content makes it beneficial for human health. It is rich in oil (52–53%) and protein (26.25%). The oil extracted from the tiny seeds is odorless, very stable, and contains an antioxidant system comprising sesamol and sesamolinol formed from sesamolin, which substantially reduce its oxidation rate. Sesame oil effectively reduces stress and tension, prevents nervous disorders and various types of cancers, relieves fatigue, and promotes strength and vitality (Nakimi, 1995; Majdalawieh et al., 2017). The global demand for vegetable oils is increasing and it is estimated to reach 240 million tons by 2050 (Barcelos et al., 2015). Sesame, high seed oil crop, may help to mitigate this growing demand. However, sesame growth and production are challenged by a number of factors, causing significant yield losses every year. Those include seed shattering, lodging, indeterminate growth, waterlogging stress, and diseases such as Phytophthora blight (PB), wilt, phyllody, powdery mildew, bacterial blight, and charcoal rot. Among these, PB is an important biotic factor in the intensive management of sesame crops in countries with excessive rainfall and humid environments.

Phytophthora blight is one of the most damaging diseases of sesame worldwide. It is caused by *Phytophthora nicotianae* Breda de Haan and occurs in sesame growing regions with moist and humid conditions. *P. nicotianae* is the dominant species in Brazil, Egypt, South Africa, and Tunisia (Panabières et al., 2016). Its optimum temperature for growth and infection is between 33 and 36°C, and it may be considered an emerging pathogen in the current context of global climate change. On the Korean Peninsula, it is the most important soil-borne disease, causing severe economic losses (Choi et al., 1987). To date, the Korean National Agrobiodiversity Germplasm has identified four *P. nicotianae* isolates, namely KACC48120, KACC48121, No2526, and No2040 (Oh et al., 2018). These strains were collected from infected sesame plants in the fields of Gyeongju, Naju, Gunwi, and Yecheon province (county) in South Korea, and artificially inoculated in sesame genotypes to evaluate PB resistance of the genotypes (Oh et al., 2018). Unfortunately, little is known about the sources of resistance, and very few efforts have been made to develop high-yielding cultivars with resistance to PB. The control measures applied in fields are often impractical and expensive. Most commercial cultivars and populations of *Sesamum* spp. lack sufficient genetic resistance that will preclude fungicide use against PB, and there is no information on the inheritance of resistance to this disease in sesame. Hence, there is a need to assess the genetic diversity among the present sesame cultivars and evaluate their resistance to *P. nicotianae.* Such studies will help to identify genetically divergent varieties that can be used in breeding (Asekova et al., 2018). Besides, these sources can be used to identify the quantitative trait loci (QTL) or markers linked to the trait controlling PB resistance.

Several molecular markers, such as simple sequence repeats (SSRs) and single-nucleotide polymorphisms (SNPs), are available in sesame owing to recent developments in next-generation sequencing (NGS) technologies. Particularly, SNP markers, which are stable, heritable, and abundant in plant genomes, are a preferred choice of markers for linkage mapping and genome-wide association studies (GWAS) (Varshney et al., 2012; Zhang et al., 2016). Genotyping-by-sequencing (GBS) has been developed as a low cost, rapid, and robust protocol for genome-wide SNP discovery and genotyping, making it an excellent tool for several crops differing in genome size, organization, and breeding system (paleopolyploids, neo-allopolyploids, neo-autopolyploids) (Elshire et al., 2011; Poland and Rife, 2012; Kim et al., 2016). The protocol includes the digestion of genomic DNA with restriction enzymes, followed by ligation of the barcode adapter, PCR amplification, and sequencing of the amplified DNA pool on a single lane of flow cells (He et al., 2014). The sequenced information is processed through several bioinformatics pipelines for further analysis and interpretation of the results. Because of its simplicity and robustness, this approach has been successfully used in high-throughput marker discovery, development of high-density linkage maps, GWAS, genomic diversity, population structure analysis, and genomic selection in several plants. In sesame, this approach has been used for high-throughput SNP identification and development of the genetic map (Uncu et al., 2016), dissection of the genetic basis for determinate growth habit (Zhang et al., 2018), and genome-wide association study of vitamin E (He et al., 2019). These studies successfully demonstrated the fitness of GBS in identifying the genetic variants in sesame populations.

In this study, we performed bulk segregant analysis and genetic mapping to identify chromosome regions governing PB resistance in two RIL populations that were evaluated against *Phytophthora nicotianae* isolates. Phenotype evaluation scores and high-density linkage map were used to identify QTLs for PB resistance. In addition, genetically diverse sesame accessions were used to carry out GWAS to identify SNPs associated with PB resistance. By comparing physical position of the SNPs identified in QTL mapping and GWAS, candidate genes were pinpointed. The results from this study will be useful in understanding the genetics of PB resistance and marker-assisted selection in future breeding programs in sesame.

## Materials and Methods

### Plant Materials, Population Development, and DNA Extraction

In this study, 87 sesame accessions were used for GWAS analysis (Supplementary Table 1). The set comprised wildtype and various known cultivars, landraces, and breeding lines of *S. alatum* and *S. indicum* originating from Korea and other countries (Supplementary Table 1). Two F_2_ segregating populations were developed containing 461 and 419 individuals by crossing a PB-resistant *S. indicum* ‘Goenbaek’ (Kim et al., 2018) with PB-susceptible *S. indicum* ‘Osan’ and ‘Milsung’ (Oh et al., 2018). These segregating populations were advanced to develop RIL populations. The obtained two RILs were named GO-RILs and GM-RILs. The cultivar Goenbaek was derived from a cross of Sungbun (a variety obtained by mutation with gamma rays) and SIG950006-4-1-1 (crossing blocks) by the breeding team at the Rural Development Administration (RDA), National Institute of Crop Science of Republic of Korea. In detail, Goenbaek was crossed with Osan and Milsung in 2016 at the Department of Southern Area Crop Science Breeding Station, National Institute of Crop Science, Miryang, South Korea. The F_1_ seeds were planted and self-pollinated in a greenhouse during the winter to produce F_2_ progeny. In 2017–18, the F_2_ seeds were planted in the greenhouse and self-pollinated to produce F_2__:__3_ family seeds, which were advanced to the F_5_ generation by single-seed descent. Finally, the F_5__:__7_ (Goenbaek × Osan; GO-RIL) and F_5__:__7_ (Goenbaek × Milsung; GM-RIL) mapping populations were developed comprising 90 and 188 lines, respectively. Genomic DNA was extracted from young leaves using modified cetyltrimethylammonium bromide (CTAB) extraction method described by Paterson et al. (1993) with some modifications to the components of the CTAB buffer to eliminate the ultra-plentiful polysaccharides in sesame leaves.

### Assessment of Phytophthora Blight Resistance

Three *Phytophthora nicotianae* isolates KACC48120, KACC48121, and No2526 were selected to evaluate the resistance of sesame accessions, F_2_ segregating populations, and their two subsequent RILs. The virulence difference among these isolates and their collection sites has been described previously (Oh et al., 2018; Supplementary Table 1). The preparation of inoculum and inoculation protocol followed that described by Oh et al. (2018). The two F_2_ segregating populations Goenbaek × Osan and Goenbaek × Milsung were evaluated for resistance using only KACC48121 isolate (the most virulent isolate). The GM-RILs were evaluated using only KACC48121 isolate using five replications for each line. The GO-RILs were screened for PB resistance against all three isolates using five replications for each line. The sesame accessions (10 replications per accession) were evaluated against all the three isolates. In detail, 1-month-old seedlings were placed in water-filled plug trays and inoculated with 5 mL of zoospore suspension (1 × 10^5^ zoospores/mL) by soil drenching method (Oh et al., 2018). The seedlings were evaluated for resistance and susceptibility 14 days after post inoculation using a 0 to 9 disease resistance scale, where 0–3 was considered resistant (R), 3.1–5 moderately resistant (MR), 5.1–7 moderately susceptible (MS), and 8–9 susceptible (S) (Oh et al., 2018).

### Bulk Segregant Analysis

Bulk segregant analysis (Michelmore et al., 1991) was used to identify the SSR markers linked to PB. Equal concentrations of DNA from 10 PB-resistant plants and 10 PB-susceptible plants were pooled to form PB-resistant bulk and a PB-susceptible bulk, respectively. The SSR marker sequences were downloaded from SisatBase^1^, and primers were synthesized by Bioneer (Daejeon, South Korea). The SSRs were first assessed for parental polymorphism, and the polymorphic markers were used to screen the resistant and susceptible bulks. A 20 μL PCR reaction mixture consisting of 50 to 100 ng of genomic DNA, 1 × reaction buffer (including 25 mM MgCl_2_), 100 μM dNTPs, 10 pmol/μL forward and reverse primers, and 0.5 U *e-Taq* DNA polymerase. All the chemicals used were purchased from SolGent Co., Ltd. (Daejeon, South Korea). PCR was performed in a DNA thermocycler (Mastercycler Nexus GSX 1; Eppendorf, Hamburg, Germany) using the following cycling parameters: 94°C (5 min); 35 cycles of 94°C (30 s), 55°C (30 s), 72°C (1 min); and a final extension at 72°C (5 min). The PCR products were stained with a DNA LoadingSTAR marker (DyneBio, Gyeonggi-do, South Korea) and analyzed by electrophoresis on 1.5% agarose gel. The PCR products were also run on a QIAxcel capillary gel electrophoresis system according to the manufacturer’s instructions (Qiagen, Hilden, Germany), and the size of each fragment was scored using the QIAxcel ScreenGel software (Qiagen, Germany). The BSA-identified polymorphic markers were then used to screen the individual F_2_ plants of each bulk, as well as the 90 GO-RILs and 188 GM-RILs, for further validation of the markers linked to PB resistance. A goodness-of-fit to the Mendelian segregation ratio was calculated using chi-square (χ^2^) analysis to examine the segregation patterns of the phenotypes and selected SSR markers.

### Genotyping-by-Sequencing and SNP Calling

The DNA was digested using the *ApeK1* restriction enzyme as described by Elshire et al. (2011). To construct a sequence library, the digested DNA was subjected to adaptor ligation, pooling, DNA purification, PCR, and quality check by agarose gel electrophoresis. The library was sequenced on the Illumina HiSeq 2000 platform by SEEDERS Co. (Daejeon, South Korea). After sequencing the samples, short reads were demultiplexed and barcode and adapter sequences eliminated. The short reads were trimmed using the Dynamic Trim and Length Sort command of SolexaQA v.1.13 (Cox et al., 2010). The cleaned reads were mapped to the reference genome *S. indicum* v.2 using the Burrows-Wheeler Alignment (BWA) tool (Li and Durbin, 2009) and the SEEDERS in-house script (Kim et al., 2014). Using the varFilter command, SNPs were called only for variable positions with a minimum mapping quality (-Q) of 30. The minimum and maximum read depths were set to 3 and 100, respectively. In-house script considering biallelic loci was used to select a significant site in the called SNP positions. Depending on the ratio of SNP/InDel reads to mapped reads, variant types were classified into three categories: homozygous SNP/InDel for more than 90%, heterozygous SNP/InDel for more than 40% and less than 60%, and the rest of them were miscellaneous. To control the quality of markers, missing proportion < 0.3 and minor allele frequency > 0.05 were selected.

### Linkage Mapping and QTL Analysis

The genotype information of the polymorphic markers (SNP and SSR) for the 90 GO-RILs was used to construct a linkage map with JoinMap 4.0 (van Ooijen, 2006). The grouping mode was set as the independent limit of detection, the mapping algorithm was used to perform regression mapping (recombination frequency < 0.4, limit of detection > 2.5, and jump = 5), and the map distances were calculated using the Kosambi map function (Kosambi, 2016). Independent limit of detection and maximum likelihood algorithms were used for grouping and ordering of markers, respectively. The ordering of the markers within each chromosome was based on the recombination events between the markers. Linkage groups and locus order were compared with published sesame linkage maps (Wang et al., 2015, 2016). Composite interval mapping (CIM) method implemented in Windows QTL Cartographer 2.0 (Wang et al., 2011) was used to identify the QTLs. A forward-selection backward-elimination stepwise regression procedure was used to identify cofactors for CIM. A 10 cM scan window was used for all analyses. The permutation tests were performed using 1,000 iterations to determine significant logarithm of odds (LOD) thresholds. Furthermore, the additive effects and phenotypic variations explained (R^2^,%) were obtained by performing CIM analysis. The QTLs with LOD > 3 were considered significant and were graphically presented on the linkage group using MapChart 2.2 (Voorrips, 2002).

### Population Structure and Phylogenetic Analyses

Population structure was assessed using the Bayesian model-based clustering technique implemented in STRUCTURE software v. 2.3.4 (Pritchard et al., 2000). The admixture model was employed with 10,000 replicates for burn-in and Markov Chain Monte Carlo iteration. Ten runs were performed for each value of clusters (*K*) ranging from 1 to 10. The optimal *K-*value was imputed from the log probability of the data likelihood [LnP(D)] and delta-K (Δ*K*) (Evanno et al., 2005). These values were then calculated with Structure Harvester (Earl, 2012). A neighbor-joining tree based on pairwise genetic distance between SNPs was constructed using Power Marker version 3.25 (NC State University, Raleigh, NC, United States) and MEGA6 (Tamura et al., 2013) software.

### Genome-Wide Association Analysis

After filtering, 8,883 SNPs covering the whole genome with minor allele frequency > 0.03 were used for GWAS analysis. Before fitting the model, each marker was coded with the value 0 for the reference allele and the value 1 for the alternative allele. Association analysis was performed for principal component analysis (PCA) and Kinship matrices based on the Compressed Mixed Linear Model (CMLM). The CMLM was implemented using the *R* package Genomic Association and Prediction Integrated Tool (GAPIT) 2.0 (Lipka et al., 2012) for association mapping. Significance of probabilities generated in the association runs was transformed by -log_10_ (*p*) (false discovery rate [FDR] *p*-value < 0.05). Scores for an individual chromosome were then inspected in Manhattan plots to determine whether the SNPs reached the significance threshold. After multiple test Bonferroni correction, significance was defined at a uniform threshold of *p* < 5.02 × 10^–7^ (-log_10_ (*p*) > 6).

### Real-Time Quantitative Reverse Transcription PCR

Real-time quantitative reverse transcription PCR (qRT-PCR) was performed for five selected resistance (*R*) genes (Supplementary Table 6) identified through the linkage and association analysis. The gene-specific primers (Supplementary Table 7) were designed based on the 5′ untranslated regions (UTR) and 3′ UTR for the gene models *SIN_1019016* (LOC110012696), *SIN_1019013* (LOC105171888), *SIN_1019001* (LOC105171879), *SIN_1018990* (LOC105172053), and *SIN_1018970* (LOC105171860). The RNA was extracted from sesame plants at different time points after inoculation using an RNeasy PowerPlant Kit (Qiagen, Valencia, CA, United States) following the manufacturer’s instructions. Comparisons of gene expression in sesame samples were made based on a time series after sesame inoculation with *P. nicotianae* KACC48121 in the two genotypes, susceptible (Milsung and Osan) and resistant (Goenbaek), collected at 2, 8, 16, 24, 36, and 48 h post-inoculation. Three biological replicates per line were used for expression analysis. Each biological replicate consisted of a pool of three stems, from three plants of each replication. Plant tissues (5 cm) were cut just below the necrotic tissue from each stem, immediately frozen in liquid nitrogen and stored at −80°C until RNA extraction. For each of these time points the equivalent control (0 h) of un-inoculated plant/tissue was also sampled for total RNA extraction and qRT-PCR analysis. First-strand cDNA was synthesized from 1 μg of total RNA using an Oligo(dT) RNA to cDNA EcoDry Premix (Takara, Dalian, China). Primers used for qRT-PCR were designed using the Primer 3Plus online software^2^ based on their DNA sequences retrieved from the sesame reference genome database at the National Center for Biotechnology and Information (NCBI) and optimized using Primer Premier 5.0 (Premier Biosoft International, Palo Alto, CA, United States). The qRT-PCR reactions were conducted as described by the manufacturer of the RT system. Reaction mixtures (20 μL) contained the cDNA reverse transcription solution (1 μL), PCR Master Mix (SYBR Green; Life Technologies LTD., Warrington, United Kingdom), and 0.2 μM of each primer.

The qRT-PCR thermocycling program consisted of one cycle at 50°C for 2 min, one cycle at 95°C for 10 min, and 40 cycles of 10 s at 95°C, 10 s at 60°C, and 30 s at 72°C. The melting curve temperature profile was obtained by heating to 95°C for 15 s, cooling to 60°C for 1 min, and slowly heating to 95°C for 15 s at 0.5°C increment every 10 s, with continuous measurement of fluorescence at 520 nm. An 18S rRNA gene was used as an internal control. All reactions were performed in triplicate, including three non-template reactions as negative controls. Gene expression was quantified using a 7900 Real-Time PCR System (Applied Biosystems, Foster City, CA, United States). Threshold values generated from the ABI PRISM 7900 Software Tool (Applied Biosystems, United States) were employed to quantify relative gene expression based on the ΔC_T_ method using a reference gene (*actin*) (Livak and Schmittgen, 2001). Each sample comprised three technical replicates, and the qRT-PCR data were analyzed with the 2^–ΔΔCt^ method (Livak and Schmittgen, 2001).

## Results

### Phenotypic Evaluation

**FIGURE 1:**
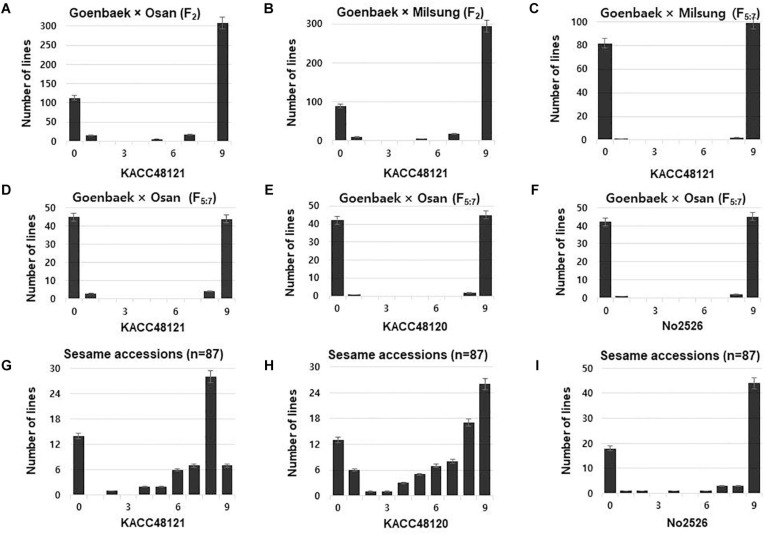
Frequency distribution of disease severity scores at 14 days post-inoculation for the F_2_, recombinant inbred lines (RILs), and sesame accessions against *Phytophthora nicotianae* isolates KACC48121, KACC48120, and No2526. **(A–C)** Goenbaek × Osan F_2_, Goenbaek × Milsung F_2_, and Goenbaek × Milsung F_5__:__7_ RILs with isolate. **(D–F)** Goenbaek × Osan F_5__:__7_ RILs. **(G–I)** Genome-wide association study (GWAS) sesame accessions. Disease severity scores: 0–3 resistant, 3.1–5 moderately resistant, 5.1–7 moderately susceptible, and 7.1–9 susceptible.

Parental cultivars (Goenbaek, Osan, and Milsung), and the two F_2_ populations of the crosses Goenbaek × Osan (GO), and Goenbaek × Milsung (GM) were evaluated for PB resistance to the KACC48121 isolate in greenhouse conditions using 0–9 disease resistance scale as described by Oh et al. (2018). Among the F_2_ plants from the cross between Goenbaek and Osan (GO), 128 plants were resistant whereas, 333 plants were susceptible, segregating in the ratio of 1:3 at χ^2^ = 2.07 and *P* = 0.15 (Figure 1A and Supplementary Table 2). Similarly, among the F_2_ plants from the cross between Goenbaek and Milsung (GM), 92 were resistant whereas, 327 were susceptible, segregating in the ratio of 1:3 at χ^2^ = 1.88 and *P* = 0.17 (Figure 1B and Supplementary Table 2). The 90 GO-RILs and the parental lines were evaluated for their resistance response to all the three *P. nicotianae* isolates. Of the 90 GO-RILs, 45 were resistant and 45 were susceptible to KACC48121 isolate. Similarly, 43 were resistant and 47 were susceptible to KACC48120 isolate, whereas, 43 were resistant and 47 were susceptible to No2526 isolate (Figures 1D–F and Supplementary Figure 1B). Both the RIL populations showed a 1:1 ratio of resistant to susceptible plants (*P* < 0.05 and *P* < 0.01) (Figures 1C–F and Supplementary Table 3).

For GWAS, 87 sesame accessions were evaluated for their resistance response to all the three *P. nicotianae* isolates. Of the 87 accessions, 20 were resistant, 5 were moderately resistant, 12 were moderately susceptible, and 50 were susceptible to *P. nicotianae* isolate KACC48121. Similarly, 19 accessions were resistant, 2 were moderately resistant, 10 were moderately susceptible, and 52 were susceptible to KACC48120 isolate; and 13 accessions were resistant, 1 was moderately resistant, 3 were moderately susceptible, and 52 were susceptible to No2526 isolate (Figures 1G–I and Supplementary Table 1).

### Bulk Segregant Analysis

To identify the SSR markers linked to PB resistance, BSA was performed using progenies from two segregating F_2_ populations. In total, 54 SSRs from chromosome 10 were used to detect polymorphisms among the parental lines and resistant and susceptible lines that were screened against KACC48121 isolate (Supplementary Table 5). Of the 54 markers, five markers (SiSSM-84917, SiSSM-84958, SiSSM-84996, SiSSM-85023, and SiSSM-86906) were polymorphic in the F_2_ plants from the cross Goenbaek × Osan, and six markers (SiSSM-84917, SiSSM-84958, SiSSM-85023, SiSSM85354, SiSSM-85570, and SiSSM-86906) were polymorphic in F_2_ plants from the cross Goenbaek × Milsung (Supplementary Figure 2A and Supplementary Tables 3, 5). Four markers were found to be polymorphic in both populations (Supplementary Figure 2B and Supplementary Tables 3, 5). These markers were individually screened against the GO-RILs and GM-RILs genotypes to verify whether the resistant and susceptible alleles co-segregate with the respective phenotype. All the linked markers displayed co-segregation with the phenotypic disease evaluation scores in GO-RILs and GM-RILs (Supplementary Table 3), which confirmed strong linkage of the markers with PB trait. The flanking sequences of these markers were retrieved from the sesame genome database and compared with the physical positions of the QTLs. These markers shared the physical positions with QTLs detected in the present study (data not shown).

### Genotyping-by-Sequencing and Linkage Map Construction

The 90 F_5__:__6_ RIL population (GO-RILs) and their parental lines (3 replications) were genotyped using GBS. The GBS analysis yielded 40.2 Gbp of raw sequence reads. The average number and total length of raw reads for each sample were 4,228,098 and 0.43 Gbp, respectively^3^ (ID NV-0660-000001, NN-6717-000001). After trimming the barcode and adaptor sequence from demultiplexed sequences of the 96 samples and removing the low-quality reads, an average number of 3,039,597 trimmed reads with a total length of 285.7 Mb was obtained; 71.94% of the total raw data mapped to the reference genome *Sesamum indicum*.

**FIGURE 2:**
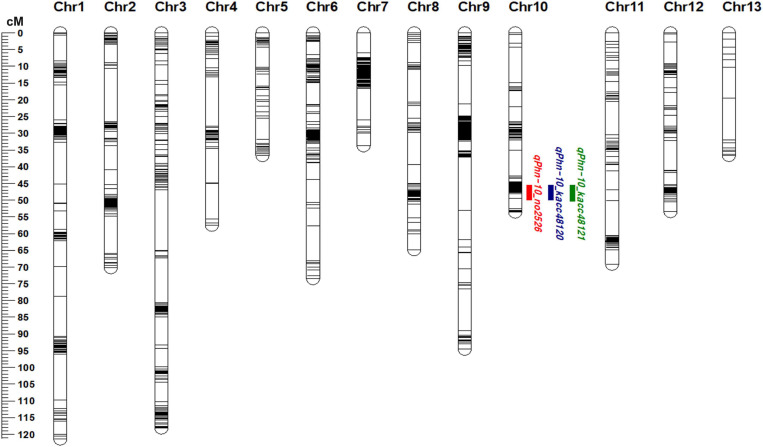
Linkage map of sesame chromosomes and quantitative trait loci (QTLs) detected using Goenbaek × Osan F_5__:__7_ recombinant inbred lines (GO-RILs) on chromosome 10. Green, blue, and red colors represent the QTLs detected against *Phytophthora nicotianae* isolates KACC48121, KACC48120 and No2526, respectively.

The markers showing more than 80% of missing data and extreme segregation were removed before linkage map construction. In total, 1662 polymorphic markers (1657 SNPs and 5 SSRs) were used for developing the linkage map. The genetic map comprised 13 chromosomes (linkage groups, LG) covering a total length of 883.37 cM (Figure 2 and Supplementary Table 4). The genetic length of the LG ranged from 33.77 cM (for LG 7) to 121.39 cM (for LG 1) (Figure 2 and Supplementary Table 4). The average genetic distance between neighboring markers was 4.69 cM (Supplementary Table 4).

### QTL Analysis

Composite interval mapping was performed to identify the QTLs (LOD > 3.0) for resistance to *P. nicotianae* isolates KACC48121, KACC48120, and No2526 using 90 GO-RILs. A total of three QTLs were detected on chromosome 10, with R^2^ ranging from 12.79 to 13.34% and LOD score ranging from 3.64 to 3.72 (Figure 2 and Table 1). The QTL *qPhn-10_kacc48121* positioned at 48.11 cM, explained 12.79% of R^2^ with LOD score 3.64 (Figure 2 and Table 1). The QTL *qPhn-10_kacc48120* positioned at 48.11 cM, explained 13.34% of R^2^ with LOD score 3.72 (Figure 2 and Table 1). The QTL *qPhn-10_no2526* positioned at 48.11 cM, explained 13.34% of R^2^ with LOD score 3.72 (Figure 2 and Table 1). All the three QTLs *qPhn-10_kacc48121*, *qPhn-10_kacc48120*, and *qPhn-10_no2526* were located between flanking markers S10_12912718 and S10_14927412 (Table 1), corresponding to physical positions 12.9 and 14.9 Mb, respectively, in the updated sesame genome^4^.

**TABLE 1:** Quantitative trait loci (QTLs) associated with PB resistance in GO-RILs for three *P. nicotianae* isolates.

**Trait**	**QTL^a^**	**Left marker**	**Right marker**	**Position (cM)**	**Physical position (bp)**	**LOD^b^**	**R^2^ (%)^c^**	**Add^d^**
KACC48121	*qPhn-10_kacc48121*	S10_12912718	S10_14927412	48.11	12912718 - 14927412	3.64	12.79	−0.38
KACC48120	*qPhn-10_kacc48120*	S10_12912718	S10_14927412	48.11	12912718 - 14927412	3.72	13.34	−0.38
No2526	*qPhn-10_no2526*	S10_12912718	S10_14927412	48.11	12912718 - 14927412	3.72	13.34	−0.38

*^*a*^Identified QTLs with the given unique ID. ^*b*^Logarithm of odds ratio at the position of the peak. ^*c*^Percent of phenotypic variance explained by the QTL. ^*d*^Additive effect of QTL.*

### Population Structure and Phylogenetic Analysis

**FIGURE 3:**
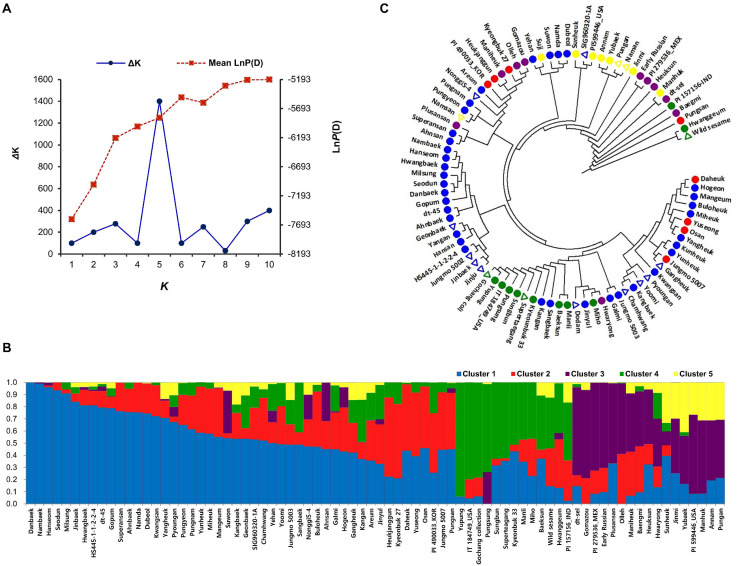
Population structure and genetic diversity of sesame accessions inferred using 8,883 single nucleotide polymorphisms. **(A)** Delta *K-*values calculated by Evanno’s method. **(B)** Classification of sesame accessions using STRUCTURE v. 2.3.4 software. **(C)** Neighbor-joining phylogenetic tree of sesame accessions. Triangles indicate resistant sesame accessions.

The sesame accessions were genotyped using GBS and the SNP information was used for population structure and phylogenetic analysis. GBS using the *ApeK1* restriction enzyme generated 575,712 SNPs. The GBS generated data is available in National Agricultural Biotechnology Information Center (NABIC^5^, ID = NV-0650, NN-7158). The SNPs with < 5% MAF were excluded as a final filtering step to call the genome-wide SNPs. Subsequently, we classified the GBS data into three groups: homozygous SNPs with a read depth of ≥ 90%, heterozygous SNPs with a read depth of ≤ 40% but ≤ 60%, and SNPs that could not be distinguished; the third group was categorized as miscellaneous. This step resulted in a set of 8,883 high-quality SNPs, which were uniformly distributed across the 13 sesame chromosomes, and their density per SNP ranged from 2.03 for chromosome 9 to 7.07 kb for chromosome 1 (the average was 3.68 kb). These results suggest that the density of these SNP sets was sufficient to capture the genetic variation in the sesame accessions for GWAS. The number of SNPs per chromosome ranged from 444 (chromosome 13) to 1,229 (chromosome 3), with an average of 684 SNPs. Population structure analysis was performed to understand the structure matrix, which is important in GWAS analysis. The results showed that the value of LnP(D) increased continuously from *k* = 1 to 10, and the total number of subpopulations was determined based on Δ*K*. The sesame accessions were divided into five distinct genetic clusters and some admixtures, shown by different colors (*K* = 5) (Figures 3A,B). The PCA (Supplementary Figure 3) and a neighbor-joining tree (Figure 3C) based on pairwise genetic distances between SNPs similarly identified several major and minor clusters (subgroups). Phylogenetic analysis placed the 16 highly resistant accessions across the phylogenetic tree, revealing the genetic diversity of sesame accession (Figure 3C).

### GWAS

The GWAS identified 29 significant SNPs associated with resistance to the three *P. nicotianae* isolates on chromosome 10 (Table 2 and Supplementary Figure 4). Of those, 13 SNPs were common for all the three isolates, three were common for two isolates (KACC48121 and KACC48120), and nine were unique to KACC48121 isolate and four to No2526 isolate, with the -log_10_(*p*) values ranging from 5.02 to 8.31 (Table 2 and Supplementary Figure 4). The explained phenotypic variation of these common SNPs ranged between 35.57 and 70.32%, and may be highly correlated to alleles conferring PB resistance as referenced by QTL mapping.

**TABLE 2:** The SNP markers (above the threshold -log_10_ *p*-values) significantly associated with PB resistance for three *P. nicotianae* isolates in sesame.

**Corresponding SNP(s)**	**SNP Position (Chr:position)^a^**	**Isolate**	**Allele**	***p-*value^b^**	**-log_10_^c^**	**R^2^ (%)^d^**
S10_14318714	chr10:14318714	KACC48121	A/G	9.59E-06	5.02	35.57
S10_14318716	chr10:14318716	KACC48121	G/C	9.59E-06	5.02	35.57
S10_14438712	chr10:14438712	KACC48121	A/T	9.59E-06	5.02	35.57
S10_14456135	chr10:14456135	KACC48121	G/A	3.25E-05	5.13	36.64
S10_14532795	chr10:14532795	KACC48121	C/T	2.33E-06	5.63	40.35
		KACC48120		5.91E-06	5.23	41.42
S10_14563939	chr10:14563939	KACC48120	T/A	1.95E-07	6.71	57.85
		KACC48121		1.60E-08	7.79	64.28
S10_14643881	chr10:14643881	KACC48121	T/C	9.59E-06	5.02	35.57
S10_14665280	chr10:14665280	KACC48121	C/A	9.59E-06	5.02	35.57
S10_14841897	chr10:14841897	KACC48120	C/G	8.83E-07	6.05	49.84
		KACC48121		1.96E-07	6.71	52.33
S10_14860890	chr10:14860890	No2526	A/G	9.48E-06	5.02	36.17
		KACC48120		1.95E-07	6.71	57.85
		KACC48121		1.60E-08	7.79	64.28
S10_14860891	chr10:14860891	No2526	G/A	9.48E-06	5.02	36.17
		KACC48120		1.95E-07	6.71	57.85
		KACC48121		1.60E-08	7.79	64.28
S10_14860929	chr10:14860929	No2526	C/G	2.27E-06	5.64	39.84
		KACC48120		5.81E-08	7.24	64.60
		KACC48121		4.88E-09	8.31	70.32
S10_14860930	chr10:14860930	No2526	T/A	2.27E-06	5.64	64.60
		KACC48120		5.81E-08	7.24	39.84
		KACC48121		4.88E-09	8.31	70.32
S10_14880164	chr10:14880164	No2526	C/T	6.49E-06	5.19	38.78
		KACC48120		8.21E-06	5.09	37.13
		KACC48121		8.04E-06	5.09	36.29
S10_14922487	chr10:14922487	No2526	A/G	1.81E-06	5.74	40.44
S10_14927344	chr10:14927344	No2526	A/G	5.66E-06	5.25	58.66
		KACC48120		4.37E-06	5.36	42.23
		KACC48121		5.96E-06	5.22	37.51
S10_14927451	chr10:14927451	No2526	A/G	4.94E-06	5.31	37.82
S10_14934271	chr10:14934271	No2526	T/C	3.02E-06	5.52	39.09
S10_14934878	chr10:14934878	KACC48121	C/T	3.82E-06	5.42	39.34
S10_14972452	chr10:14972452	KACC48121	A/G	5.87E-06	5.24	37.63
S10_15011585	chr10:15011585	No2526	C/A	9.12E-07	6.04	44.26
		KACC48120		2.66E-06	5.58	42.26
		KACC48121		4.28E-07	6.37	48.79
S10_15019593	chr10:15019593	KACC48121	C/T	2.60E-06	5.59	40.96
S10_15031344	chr10:15031344	No2526	A/C	9.21E-07	6.04	58.66
		KACC48120		1.68E-07	6.77	37.47
		KACC48121		2.38E-08	7.62	37.51
S10_15081876	chr10:15081876	No2526	C/A	1.36E-06	5.87	41.19
		KACC48120		1.94E-06	5.71	45.84
		KACC48121		1.71E–06	5.77	42.72
S10_15091261	chr10:15091261	No2526	T/C	5.66E-06	5.25	37.47
		KACC48120		4.37E-06	5.36	41.82
		KACC48121		5.96E-06	5.22	37.51
S10_15098669	chr10:15098669	No2526	A/T	9.48E-06	5.02	36.17
		KACC48120		1.95E-07	6.71	57.85
		KACC48121		1.60E-08	7.79	64.28
S10_15109810	chr10:15109810	No2526	C/A	5.66E-06	5.25	37.47
		KACC48120		4.37E-06	5.36	41.82
		KACC48121		5.96E-06	5.22	37.51
S10_15109872	chr10:15109872	No2526	T/C	6.49E-06	5.19	37.13
		KACC48120		8.21E-06	5.09	38.78
		KACC48121		8.04E-06	5.09	36.29
S10_15109921	chr10:15109921	No2526	G/A	1.03E-06	5.99	41.94

*^*a*^Chromosome and position of marker was determined according to *Sesamum indicum* (version 2.0) (http://ocri-genomics.org/Sinbase_v2.0). ^*b*^FDR: False discovery rate. FDR Adjusted *p-*values were calculated by GAPIT applying the Benjamini-Hochberg (1995). ^*c*^FDR-controlling after Bonferroni correction was applied (*P* = 0.0001) procedure. ^*d*^Phenotypic variations explained by the significant SNPs detected by GWAS.*

### Pinpointing Candidate Genes

**FIGURE 4:**
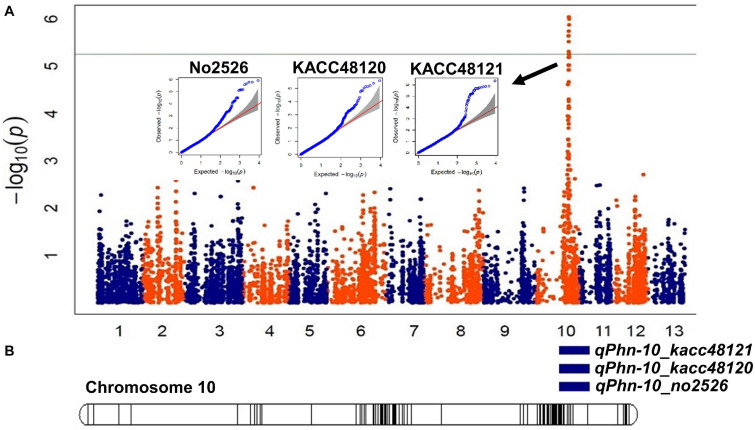
Identification of genomic region associated with Phytophthora blight (PB) resistance using an integrated approach of QTL mapping and genome-wide association study (GWAS). **(A)** Manhattan plots depicting single nucleotide polymorphisms (SNPs) significantly associated with resistance to *Phytophthora nicotianae* isolates KACC48121, KACC48120, and No2526. Each dot represents a SNP. The significant threshold was adjusted at –log_10_(*p*) > 5.02. **(B)** A linkage map of chromosome 10 showing significant QTLs identified for PB resistance in Goenbaek × Osan F_5__:__7_ recombinant inbred lines (GO-RILs).

The physical positions of the significant SNPs detected by GWAS suggested that these SNPs are positioned within the intervals of the QTLs detected in GO-RILs (Figures 4A,B). The position of the flanking markers suggested that the QTLs were located within a 2.01 Mb interval (12,912,718–14,927,412 bp) (Figure 4B and Table 1), which was found to colocate with the chromosomal positions of GWAS-SNPs (S10_14318714 and S10_15109921) (Table 2). These SNPs spanned a distance of 0.791 Mb (14.31–15.10 Mb) on chromosome 10. Since this region was detected in both GWAS analysis and QTL mapping, it was further targeted for identifying the possible candidate R-genes (Supplementary Table 6). Nineteen out of 29 SNPs were detected in the intergenic regions; however, no SNPs were detected in the coding region.

**FIGURE 5:**
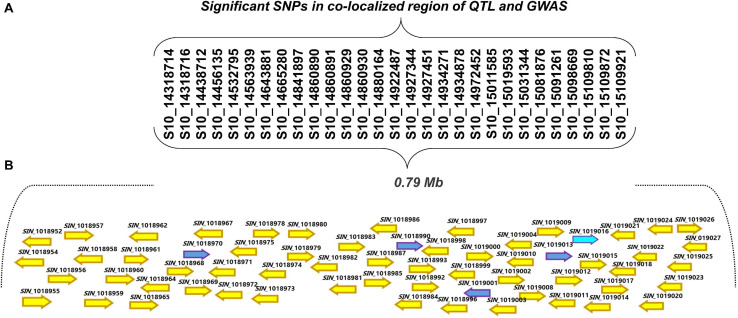
**(A)** Significant single nucleotide polymorphisms (SNPs) within the 0.79 Mb region found to be associated with PB resistance by GWAS and QTL mapping. **(B)** An open reading frames of the gene models present in the 0.79 Mb region on chromosome 10 controlling PB resistance in sesame. Blue arrows represent the genes that were selected for the expression analysis.

The corresponding genomic sequences of the QTL-GWAS co-located region were retrieved from the sesame genome database. An annotation analysis showed that chromosome 10 from 14.31 to 15.10 Mb associated with 29 SNPs contained 80 genes (Figures 5A,B and Supplementary Table 6). These genes, starting from the letter “*SIN*,” were described according to the *S. indicum* v.2 reference database^6^. Each SNP in the intergenic position was considered for potential functional annotation based on the actual proximity of nearby located genes (Table 2 and Supplementary Table 6). Thirty-four putative genes were present in the corresponding genomic region. Of these, nine genes encoding the NB-ARC domain (also identified as nucleotide binding site leucine-rich repeat [NBS-LRR] proteins) found in many defense-related genes (Supplementary Table 6), eight genes encoded protein kinase-like and Ser/Thr protein phosphatases, and three genes encoded pentatricopeptide repeat-containing protein domains, both of which are stress-associated proteins (Supplementary Table 6; Takemiya et al., 2013; Fu et al., 2020).

### Expression Patterns and Sequencing Analysis of the Candidate Genes

**FIGURE 6:**
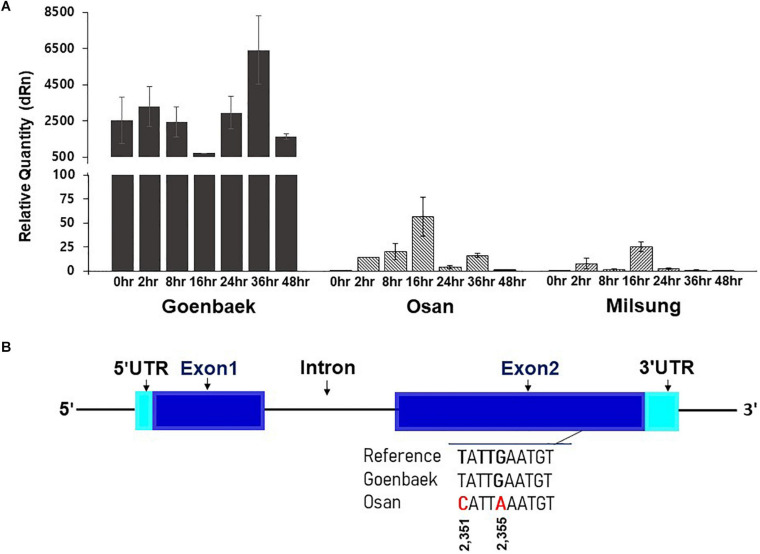
**(A)** The real-time quantitative reverse transcription PCR (qRT-PCR) analysis of the *SIN_1019016* (homolog of *At1g58390*) gene at seven time-points (0, 2, 8, 16, 24, 36, and 48 h) after inoculation of KACC48121 in Goenbaek, Osan, and Milsung sesame cultivars. At each time point, expression values are the average of three biological replicates normalized against the mean of reference gene (*actin*) as endogenous control; vertical bars indicate standard deviation. **(B)** The gene structure of *SIN_1019016*. Blue rectangles indicate exons, black line indicates the intron, and turquoise rectangles indicate untranslated regions (UTRs). In exon 2, the single nucleotide polymorphisms (SNPs) among sesame cultivars Goenbaek and Osan and sesame reference genome sequence of Zhongzhi13. *Note*: Figure S6 presents the whole gene sequence.

Among the candidate genes identified on chromosome 10 by combined biparental QTL mapping and GWAS, we selected five candidate genes that are strongly associated with disease resistance function: *SIN_1019016* (probable homolog of disease resistance protein *At1g58390*; LOC110012696), *SIN_1019013* (proline-rich receptor-like protein kinase PERK3, LOC105171888), *SIN_1019001* (receptor-like protein kinase S.2, LOC105171879), *SIN_1018990* (putative disease resistance RPP13-like protein 1, LOC105172053), and *SIN_1018970* (vesicle-associated membrane protein 722, LOC105171860) for validation using qRT-PCR to further assess their role in PB resistance (Supplementary Table 7). No high level of relative expression was observed for the four genes, *SIN_1019013*, *SIN_1019001*, *SIN_1018990*, and *SIN_1018970* at all time points in the Goenbaek, Milsung, and Osan parent lines (data not shown). However, expression of the *SIN_1019016* (homolog of *At1g58390*) gene in the resistant line Goenbaek was higher than the Milsung and Osan lines (both susceptible). At 24, 36, and 48 h post-inoculation, gene expression was reduced in both susceptible lines. In contrast, the gene expression was significantly increased at 36 h post-inoculation in Goenbaek, the resistant parental line (Figure 6A). The gene expression was the highest in uninoculated Goenbaek, Nuri (a commercial resistant cultivar), and RIL39 (selected resistant RIL from GM-RILs), whereas the uninoculated susceptible lines Milsung, Osan, and two susceptible RILs, RIL26 and RIL34 (selected from GM-RILs), displayed no gene expression (Supplementary Figure 5). Thus, RT-PCR analysis confirmed that *SIN_1019016* (homolog of *At1g58390*) was significantly correlated with PB resistance in the resistant commercial cultivar Nuri and RIL39 (Supplementary Figure 5). The results of qRT-PCR and RT-PCR indicated the role of *SIN_1019016* in PB resistance in sesame cultivars.

To further reveal the nucleotide variation of the gene *SIN_1019016* among the parental lines, we performed paired-end NGS of two sesame cultivars Goenbaek and Osan, using Illumina HiSeq X platform at the National Institute of Agricultural Science and Technology (Jeonju-si, South Korea). The nucleotide sequences of 3,344 bp were used as a query for BLASTN searches with cut-off *E*-value of 1e^–5^ against *S. indicum* reference genome and identified to completely match with a genomic region of 14,553,980–14,557,323 bps on chromosome 10. This genomic region harbored a single gene, GeneID Zhongzhi13_25858. Sequence variations within the genomic region (chr10:14,554,076–14,557,285 bp) of the sesame gene ID Zhongzhi13_25858 were identified by searching the significant SNP list. The sequence alignment of the *SIN_1019016* (homolog of *At1g58390)* transcript revealed two synonymous SNPs between Goenbaek and Osan in exon 2 of the coding DNA sequence. These SNPs at positions 2,351 (T→C) and 2,355 (G→A) of the open reading frame caused no amino acid change (Figure 6B and Supplementary Figure 6). Further analysis of this nucleotide change and functional characterization would be necessary to reveal the role of this gene in PB resistance in sesame.

## Discussion

In the present study, we employed an integrated approach involving GWAS, biparental QTL mapping, and bulk segregant analysis to understand the PB resistance in sesame. This is the first comprehensive study that used an association panel of sesame accession, F_2_ segregating populations, and their subsequent RILs and screened them against multiple isolates of *Phytophthora nicotianae* to identify the resistance loci for molecular breeding. Molecular tagging of the gene(s) or QTLs conferring PB resistance has not been previously reported in sesame. Screening for disease-resistance loci using molecular markers enables the selection for resistance without laborious disease screening trials and facilitate breeding efforts to efficiently develop PB-resistant sesame cultivars.

Genome-wide association study detects non-random association of the alleles for a particular trait in a set of association panels, whereas biparental QTL mapping detects the phenotypic variation displayed by the alleles within the two parents contrasting for a trait of interest (Han et al., 2018). The QTL region detected by biparental mapping encompasses a large genomic region, thus impeding the development of tightly linked markers. GWAS can narrow down those QTL regions up to candidate gene level, but it can also produce a high rate of false positives, which require additional validation. The combined use of these approaches can overcome the shortcomings of both techniques (Siddique et al., 2019). Therefore, in this study, we used GWAS to detect SNPs linked to the PB resistance in a set of sesame accessions and three lines (resistant Goenbaek and susceptible Milsung and Osan) and develop mapping population to be used for QTL identification. For association and mapping studies, the high-throughput molecular markers are required to construct an ultrahigh-density linkage map and conduct linkage disequilibrium analysis (He et al., 2019). NGS technologies offer tools for rapid discovery of SNP markers. Among those technologies, the GBS method has a relatively low cost, it is simple, and can reduce the complexity of large genomes to analyze nucleotide sequences (Elshire et al., 2011; Kulkarni et al., 2018). We successfully utilized the GBS technique to generate abundant SNPs for association and mapping studies. The GBS analysis in our study detected high-quality polymorphic SNPs, 8,883 among the 87 sesame accessions and 1657 among the 90 RILs, which were then used in GWAS and biparental QTL mapping, respectively.

Low level of polymorphism in sesame genotypes has been observed during trait-linked marker developments by several researchers, which enabled the recent use of GBS for large-scale SNP identification (Uncu et al., 2016; Zhang et al., 2018). Several research groups successfully developed SNP markers using small population panels through GBS approach in sesame (Uncu et al., 2016; He et al., 2019). A recent GWAS in sesame based on 5,962 GBS-SNPs using 96 accessions revealed several useful SNP markers co-relating with high vitamin E content on chromosomes 3 and 8 (He et al., 2019). Another research group constructed a genetic linkage map in sesame with total genetic distance of 914 cM based on 15,521 GBS-SNPs obtained from 91 RILs and confirmed the linkage map accuracy by mapping the previously reported SSR markers on the same linkage map (Uncu et al., 2016; Zhang et al., 2018). The genotyping approach and population size (association panel and RILs) used in aforementioned studies were confirmed in our study when mapping the loci linked to important traits. High-throughput genotyping approaches based on NGS sequencing technologies such as GBS can facilitate the mapping of important loci using a relatively small number of association panels and mapping populations (He et al., 2019). Nevertheless, large gaps have been detected in the developed SNP-based linkage maps (Uncu et al., 2016). Similarly, we observed several large gaps in the genetic linkage maps among adjacent markers (Figure 3). However, the integrated approach implemented here, involving GWAS of divergent accessions and biparental QTL mapping supported by bulk segregant analysis using two segregating F_2_ and RIL populations, facilitated the detection of the PB-associated locus on sesame chromosome 10.

In the present study, the biparental QTL mapping using GO-RILs detected a common QTL segment on chromosome 10, explaining 13.34% (R^2^) of phenotypic variation (Table 1) for the three *P. nicotianae* isolates (KACC48121, KACC48120, and No2526), which are the dominant types in Korea (Oh et al., 2018). The QTL segment spanned a 2.01 Mb region flanked by SNP markers. Previous studies have reported the large QTL segment for resistance to *Phytophthora* spp. in solanaceous crops (Siddique et al., 2019). Simultaneously, we also performed GWAS of 89 sesame accessions for PB resistance against the same three *P. nicotianae* isolates, and detected 29 significant SNPs (-log_10_ (*p*) = 5.02–8.31) associated with the PB trait (Table 2). These SNPs spanned a distance of 0.79 Mb (Figure 4) on chromosome 10. All the SNPs were found to be located in the intergenic regions. Comparison of the physical positions of these SNPs, and those of flanking markers of the QTLs identified in this study indicated that the GWAS-SNPs were positioned between the physical positions of the flanking markers of the QTLs identified in the biparental mapping experiments.

The genomic region on chromosome 10 identified using biparental mapping and GWAS was further scanned for candidate genes within the 0.79 Mb interval. A total of 80 genes were annotated in the corresponding genomic region, 34 of which were associated with disease resistance (Figure 5B and Supplementary Table 6). These genes encoded various domains, such as NBS-LRR proteins (have a role in defense) (Takemiya et al., 2013; Fu et al., 2020), protein kinase-like and Ser/Thr protein phosphatases, pentatricopeptide repeat-containing protein domains (stress-associated proteins), ubiquitin ligases, and ubiquitin-related modifiers (play a role in the regulation of pathogen-induced signaling) (Stone, 2014), F-box proteins (interact with plant disease hormones such as jasmonic acid and promote the expression of jasmonic acid-responsive genes) (Stotz et al., 2013), and cytochrome P450 family (*R* genes) (Han et al., 2015). These genes were speculated to have a role in conferring PB resistance in sesame. To further assess their role in PB resistance, we performed an expression analysis of five selected candidate genes representing the major defense-associated gene families (Supplementary Table 7). The *SIN_1019016* (homolog of *At1g58390*) gene was highly expressed in the resistant line Goenbaek compared to its expression in both susceptible parental lines, and its expression was significantly increased at 36 h post inoculation (Figure 6A). The qRT-PCR analysis showed that the upregulation of *SIN_1019016* in the resistant line may have played a role in conferring PB resistance. Expression analysis of other genes in the QTL intervals and functional characterization may provide additional molecular information regarding the role of this genomic region in controlling PB resistance in sesame.

Nucleotide binding site-leucine-rich repeat genes are the largest class of disease resistance (*R*) genes in plants and are found as single (singletons) or in cluster of genes (tightly linked arrays of related genes) (Leister, 2004). Several *R* genes encoding NBS-LRR proteins can confer PB resistance in plants. These include the *R1* gene for late blight resistance in potato (Ballvora et al., 2002), the *Rps1* region containing PB resistance genes in soybean (Bhattacharyya et al., 2005), and the *Ph-3* gene from *Solanum pimpinellifolium* conferring resistance to *Phytophthora infestans* (Zhang et al., 2014). In our study, nine genes from the QTL-associated genomic regions encoded NBS-LRR proteins, one of which was analyzed for its expression. This study is the first in sesame to show the role of *SIN_1019016*, which encodes the NBS-LRR protein, in conferring resistance to *P. nicotianae* isolates. Apart from the *SIN_1019016* gene, other candidate genes within the QTL regions identified in this study may also have a significant role in PB resistance. Therefore, in−depth deciphering of the genetic regions associated with PB resistance detected in the present study along with expression and functional characterization of additional candidate genes in this genomic region may reveal further information related to the molecular mechanisms involved in PB resistance in sesame.

## Conclusion

In this study, we demonstrated the applicability of GBS-based GWAS analysis coupled with biparental mapping approach in the identification of QTLs and candidate genes controlling PB resistance in sesame. By combining these approaches, we were able to identify the QTLs and significant SNPs/genomic regions associated with PB resistance in sesame. Among the predicted candidate genes, one of the genes showed higher expression in a resistant parent, indicating its potential role against PB resistance in sesame. Further molecular analysis of the QTL regions and functional characterization of the candidate genes identified in this study could provide insights into the molecular basis of PB resistance in sesame. The resistance accessions detected in our studies are a valuable source for incorporating resistance in elite sesame cultivars.

## Data Availability Statement

GBS of biparental population and GBS-GWAS raw data [(fastq files in the form of NGS SRA as well as SNP (VCF)] were deposited in the National Agricultural Biotechnology Information Center (NABIC) (http://nabic.rda.go.kr) with the following accession numbers (NN-6717-00001, NN-7158-000001, NV-0650-000001, and NV-0660-000001).

## Author Contributions

SA performed the experiments, analyzed the data, and wrote the manuscript. EO conceived this project. KPK interpreted the results, critically evaluated, and revised the manuscript. MIS and J-DL signed the experiments and revised the manuscript. SA, S-UK, MK, ML, JK, T-JH, K-WO, and K-SC helped to perform the experiments and collect the data. All authors read and approved the submission of this manuscript.

## Conflict of Interest

The authors declare that the research was conducted in the absence of any commercial or financial relationships that could be construed as a potential conflict of interest.

## Footnotes

1 http://www.sesame-bioinfo.org/SisatBase/

2 http://bioinformatics.nl/cgi-bin/primer3plus

3 http://nabic.rda.go.kr/

4 http://ocri-genomics.org/Sinbase_v2.0

5 https://nabic.rda.go.kr/

6 http://www.sesame-bioinfo.org/Sinbase2.0/

## Supplementary Material

Supplementary Figure 1 Plant wilting caused by *Phytophthora nicotianae* isolate KACC48121. **(A)** Resistant (Goenbaek) and susceptible (Osan) sesame lines at 14 days post-inoculation **(B)** Resistant (Goenbaek) and susceptible (Milsung) sesame lines at 14 days post-inoculation.

Supplementary Figure 2 Identification of SSR markers linked to Phytophthora blight (PB) resistance using bulked segregant analysis (BSA). **(A)** SSR markers shown on resistance parent Goenbaek (PI), susceptible parent Milsung (P2), resistance bulk, and susceptible bulk. **(B)** SSR markers shown on resistant parent Goenbaek (PI), susceptible parent Osan (P2), resistance bulk (BS1), and susceptible (BS2) bulk. Banding pattern of the resistance parent matches with the resistance bulk, and banding pattern of the susceptible parent matches with the susceptible bulk, indicating that the SSR markers are associated with PB resistance.

Supplementary Figure 3 Principal component analysis of sesame accession based on 8,883 single nucleotide polymorphisms (SNPs).

Supplementary Figure 4 Manhattan plots based on genotyping-by-sequencing (GBS)-based genome-wide association analysis (GWAS) showing the single nucleotide polymorphisms (SNPs) significantly associated with Phytophthora blight (PB) resistance. **(A)** The SNPs significantly associated with KACC48120 isolate. **(B)** The SNPs significantly associated with KACC48121 isolate, and **(C)** The SNPs significantly associated with No2526 isolate on chromosome 10.

Supplementary Figure 5 Expression of the *SIN_1019016* (homolog of *At1g58390*) gene in sesame uninoculated lines. 1 kb DNA ladder (#1), resistant Goenbaek and Nuri (#2 and #3) cultivars, susceptible Milsung and Osan (#4 and #5) cultivars, susceptible RIL26 and RIL34 (#6 and #7) and resistant RIL39 (#8) inbred lines of GM-RILs; *actin* was used as reference gene. Each sample was analyzed using 30 cycles in the RT-PCR.

Supplementary Figure 6 Single nucleotide polymorphism (SNPs) among Goenbaek, Osan, and sesame reference genome (Zhongzhi13) sequence of *SIN_1019016.*

Supplementary Table 1 Details of the germplasm lines used in the association analysis in the present study.

Supplementary Table 2 Segregation ratio and chi-square analysis in F_2_ individuals from the cross of Goenbaek × Osan (G × O), and Goenbaek × Milsung (G × M) inoculated with KACC48121.

Supplementary Table 3 Phenotypic evaluation and segregation of the single sequence repeat (SSR) markers segregation in recombinant inbred line (RIL) populations.

Supplementary Table 4 Detailed information of the genetic map developed by using genotyping-by-sequencing (GBS)-generated single nucleotide polymorphism (SNP) markers.

Supplementary Table 5 Details of simple sequence repeat markers used for parental polymorphism survey between parental lines Goenbaek, Osan, and Milsung and two contrasting bulks G × O and G × M.

Supplementary Table 6 Candidate resistance genes from the 0.79 Mb genomic region on chromosome 10 significantly associated with genome-wide association study (GWAS) regions and QTLs for Phytophthora blight (PB) resistance and their gene ontology (GO) descriptions.

Supplementary Table 7 List of oligonucleotide primers used for qRT-PCR analysis.
