# Supplementary material for: An Integrated Approach of QTL Mapping and Genome-Wide Association Analysis Identifies Candidate Genes for Phytophthora Blight Resistance in Sesame (Sesamum indicum L.)
Source: Front Plant Sci. 2021 Feb 16;12:604709. doi: 10.3389/fpls.2021.604709 (PMC7920980; doi:10.3389/fpls.2021.604709)
Supplement: Supplementary Figure 1 — Plant wilting caused by Phytophthora nicotianae isolate KACC48121. (A) Resistant (Goenbaek) and susceptible (Osan) sesame lines at 14 days post-inoculation (B) Resistant (Goenbaek) and susceptible (Milsung) sesame lines at 14 days post-inoculation. [file Data_Sheet_1.pdf]

An Integrated Approach of QTL Mapping and Genome-Wide Association Analysis Identifies Candidate Genes for Phytophthora Blight Resistance in Sesame (*Sesamum indicum* L.)

Sovetgul Asekova[1‡], Eunyoung Oh[1‡], Krishnanand P. Kulkarni[2,4], Muhammad Irfan Siddique[3], Myoung Hee Lee[1], Jung In Kim[1], Jeong-Dong Lee[2], Minsu Kim[2], Ki-Won Oh[1], Tae-Joung Ha[1], Sungup Kim[1*] and Kwang-Soo Cho[1*]

Corresponding authors:

Cho Kwang-Soo: kscholove@korea.kr

Kim Sungup: sesameup@korea.kr

Supporting Information

SUPPLEMENTARY FIGURE S1. Plant wilting caused by *Phytophthora nicotianae* isolate KACC48121. (**A)** Resistant (Goenbaek) and susceptible (Osan) sesame lines at 14 days post-inoculation (**B)** Resistant (Goenbaek) and susceptible (Milsung) sesame lines at 14 days post-inoculation.

SUPPLEMENTARY FIGURE S2. Identification of SSR markers linked to Phytophthora blight (PB) resistance using bulked segregant analysis (BSA). **(A)** SSR markers shown on resistance parent Goenbaek (PI), susceptible parent Milsung (P2), resistance bulk, and susceptible bulk. **(B)** SSR markers shown on resistant parent Goenbaek (PI), susceptible parent Osan (P2), resistance bulk (BS1), and susceptible (BS2) bulk. Banding pattern of the resistance parent matches with the resistance bulk, and banding pattern of the susceptible parent matches with the susceptible bulk, indicating that the SSR markers are associated with PB resistance.

SUPPLEMENTARY FIGURE S3. Principal component analysis of sesame accession based on 8,883 single nucleotide polymorphisms (SNPs).

SUPPLEMENTARY FIGURE S4. Manhattan plots based on genotyping-by-sequencing (GBS)-based genome-wide association analysis (GWAS) showing the single nucleotide polymorphisms (SNPs) significantly associated with Phytophthora blight (PB) resistance. **(A)** The SNPs significantly associated with KACC48120 isolate. **(B)** The SNPs significantly associated with KACC48121 isolate, and **(C)** The SNPs significantly associated with No2526 isolate on chromosome 10.

SUPPLEMENTARY FIGURE S5. Expression of the *SIN_1019016* (homologue of *At1g58390*) gene in sesame uninoculated lines. 1 kb DNA ladder (#1), resistant Goenbaek and Nuri (#2 and #3) cultivars, susceptible Milsung and Osan (#4 and #5) cultivars, susceptible RIL26 and RIL34 (#6 and #7) and resistant RIL39 (#8) inbred lines of GM-RILs; *actin* was used as reference gene. Each sample was analyzed using 30 cycles in the RT-PCR.

SUPPLEMENTARY FIGURE S6. Single nucleotide polymorphism (SNPs) among Goenbaek, Osan, and sesame reference genome (Zhongzhi13) sequence of *SIN_1019016.*

SUPPLEMENTARY TABLE S1. Details of the germplasm lines used in the association analysis in the present study.

SUPPLEMENTARY TABLE S2. Segregation ratio and chi-square analysis in F_2 individuals from the cross of Goenbaek × Osan (G×O), and Goenbaek × Milsung (G×M) inoculated with KACC48121.

SUPPLEMENTARY TABLE S3. Phenotypic evaluation and segregation of the single sequence repeat (SSR) markers segregation in recombinant inbred line (RIL) populations.

SUPPLEMENTARY TABLE S4. Detailed information of the genetic map developed by using genotyping-by-sequencing (GBS)-generated single nucleotide polymorphism (SNP) markers.

SUPPLEMENTARY TABLE S5. Details of simple sequence repeat markers used for parental polymorphism survey between parental lines Goenbaek, Osan, and Milsung and two contrasting bulks G × O and G × M.

SUPPLEMENTARY TABLE S6. Candidate resistance genes from the 0.79 Mb genomic region on chromosome 10 significantly associated with genome-wide association study (GWAS) regions and QTLs for Phytophthora blight (PB) resistance and their gene ontology (GO) descriptions.

SUPPLEMENTATY TABLE S7. List of oligonucleotide primers used for qRT-PCR analysis.

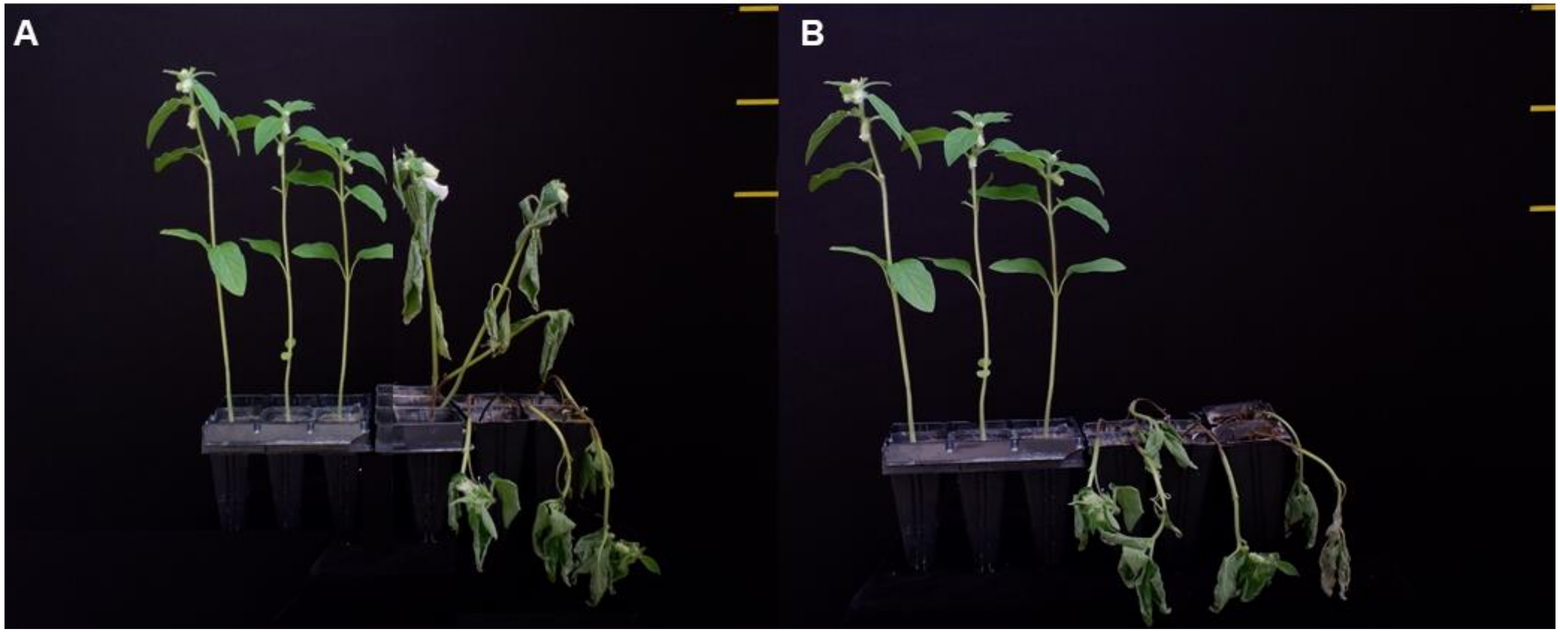

SUPPLEMENTARY FIGURE 1. Plant wilting caused by *Phytophthora nicotianae* isolate KACC48121. **(A)** Resistant (Goenbaek) and susceptible (Osan) sesame lines at 14 days post-inoculation **(B)** Resistant (Goenbaek) and susceptible (Milsung) sesame lines at 14 days post-inoculation.

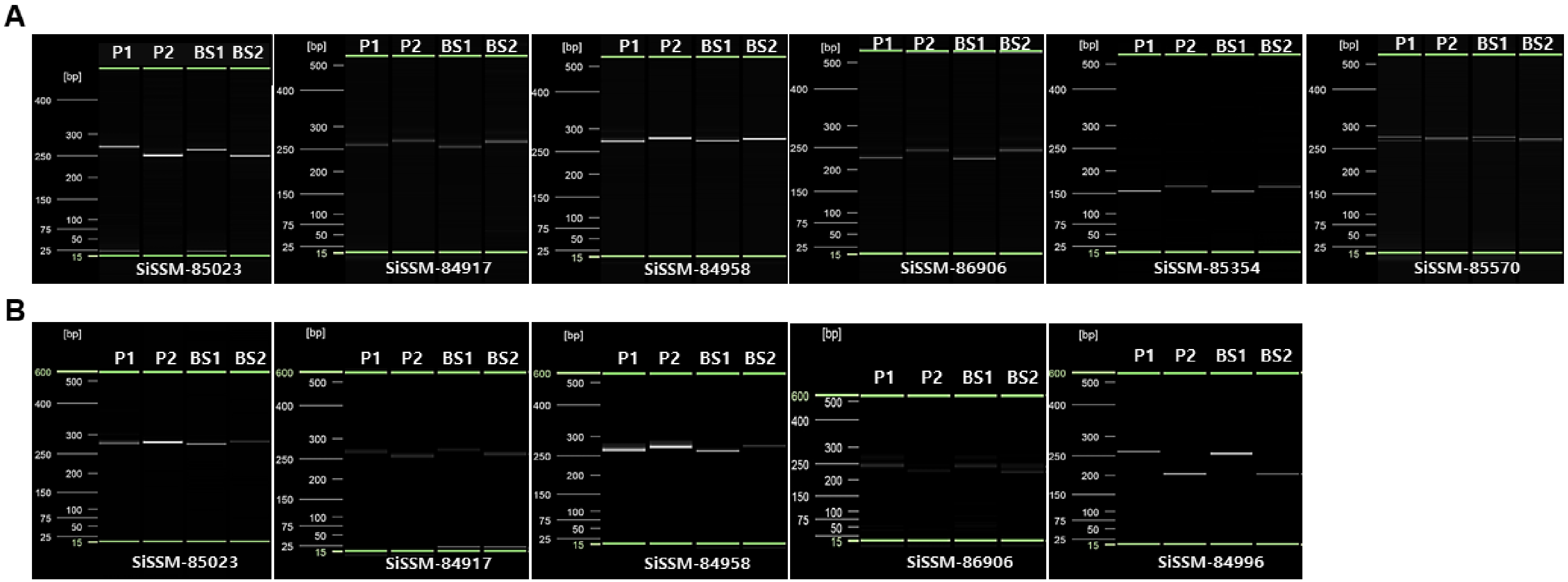

SUPPLEMENTARY FIGURE S2. Identification of SSR markers linked to Phytophthora blight (PB) resistance using bulked segregant analysis (BSA). **(A)** SSR markers shown on resistance parent Goenbaek (PI), susceptible parent Milsung (P2), resistance bulk, and susceptible bulk. **(B)** SSR markers shown on resistant parent Goenbaek (PI), susceptible parent Osan (P2), resistance bulk (BS1), and susceptible (BS2) bulk. Banding pattern of the resistance parent matches with the resistance bulk, and banding pattern of the susceptible parent matches with the susceptible bulk, indicating that the SSR markers are associated with PB resistance.

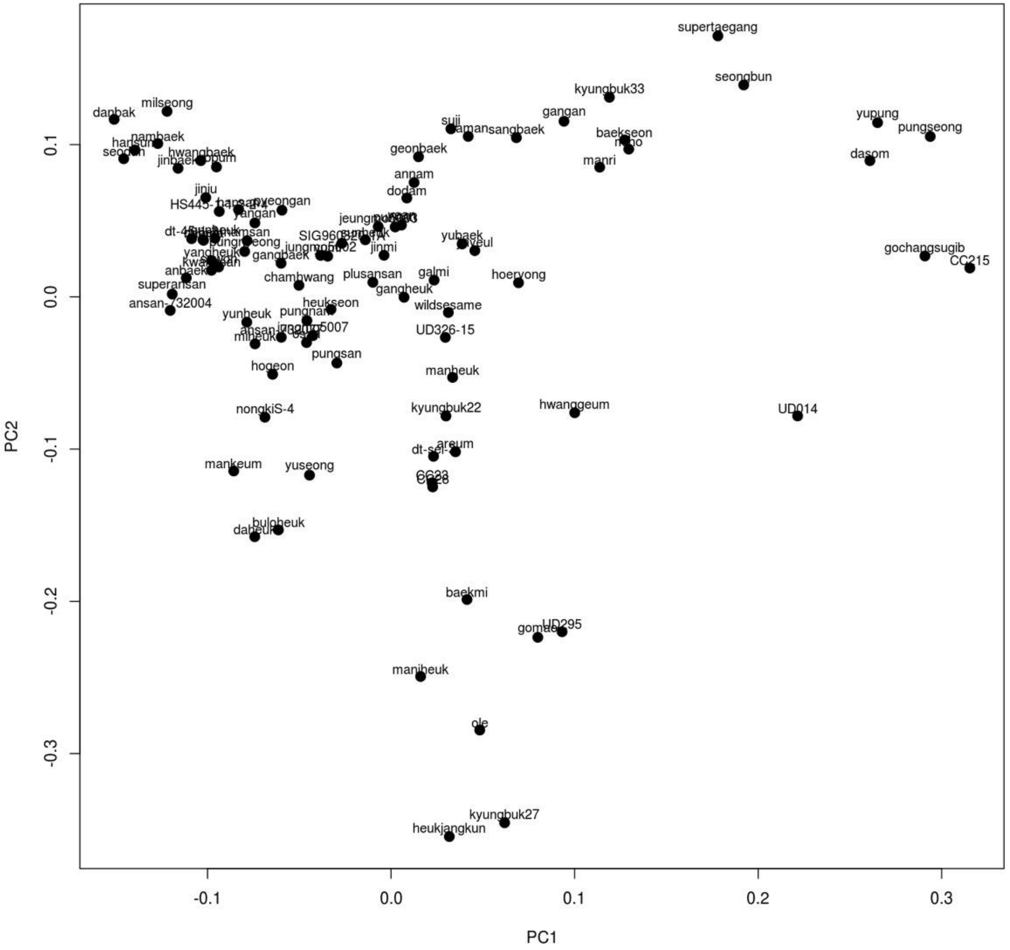

SUPPLEMENTARY FIGURE S3. Principal component analysis of sesame accession based on 8,883 single nucleotide polymorphisms (SNPs).

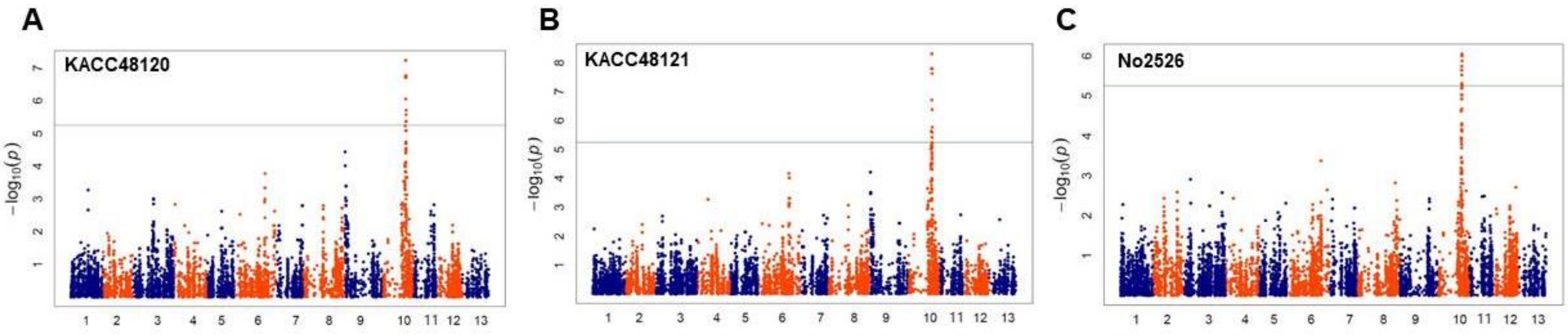

SUPPLEMENTARY FIGURE S4. Manhattan plots based on genotyping-by-sequencing (GBS)-based genome-wide association analysis (GWAS) showing the single nucleotide polymorphisms (SNPs) significantly associated with Phytophthora blight (PB) resistance. **(A)** The SNPs significantly associated with KACC48120 isolate. **(B)** The SNPs significantly associated with KACC48121 isolate, and **(C)** The SNPs significantly associated with No2526 isolate on chromosome 10.

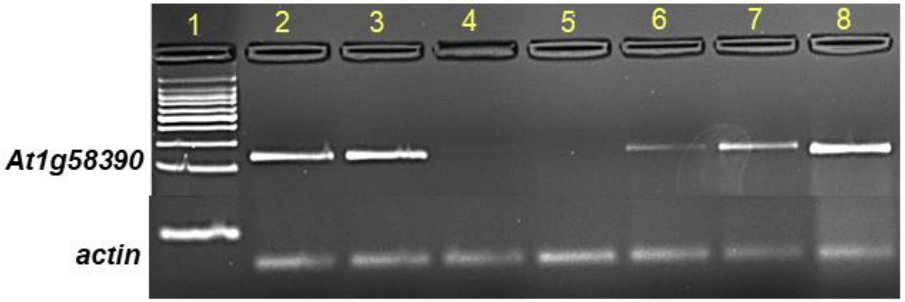

SUPPLEMENTARY FIGURE S5. Expression of the *SIN_1019016* (homologue of *At1g58390*) gene in sesame uninoculated lines. 1 kb DNA ladder (#1), resistant Goenbaek and Nuri (#2 and #3) cultivars, susceptible Milsung and Osan (#4 and #5) cultivars, susceptible RIL26 and RIL34 (#6 and #7) and resistant RIL39 (#8) inbred lines of GM-RILs; *actin* was used as reference gene. Each sample was analyzed using 30 cycles in the RT-PCR.

Reference	ATGGTTGATT	CTGTTGCAAC	AATAGCTCCT	GAAACACTTC	GGGACCTGTT	GATTGAGGAA	GCAAAGTTTT	TATCGAGTGT	GGACGGTGAG	GTTGAGGAAG	TCCGTAGGCA	GCTCAACATC	120
Goenbaek	ATGGTTGATT	CTGTTGCAAC	AATAGCTCCT	GAAACACTTC	GGGACCTGTT	GATTGAGGAA	GCAAAGTTTT	TATCGAGTGT	GGACGGTGAG	GTTGAGGAAG	TCCGTAGGCA	GCTCAACATC	120
Osan	ATGGTTGATT	CTGTTGCAAC	AATAGCTCCT	GAAACACTTC	GGGACCTGTT	GATTGAGGAA	GCAAAGTTTT	TATCGAGTGT	GGACGGTGAG	GTTGAGGAAG	TCCGTAGGCA	GCTCAACATC	120
	**********	**********	**********	**********	**********	**********	**********	**********	**********	**********	**********	**********	
Reference	ATGCACTGTT	TCTTGAAGGA	TGCTGATAGG	AGGCAAGATC	GGTACAATTC	ACAGACAGAC	CAGAATTGGG	TCGCTGAACT	TCGCGATTTG	TCCATTCAAG	CTGAGAATGT	ACTAGAAAGA	240
Goenbaek	ATGCACTGTT	TCTTGAAGGA	TGCTGATAGG	AGGCAAGATC	GGTACAATTC	ACAGACAGAC	CAGAATTGGG	TCGCTGAACT	TCGCGATTTG	TCCATTCAAG	CTGAGAATGT	ACTAGAAAGA	240
Osan	ATGCACTGTT	TCTTGAAGGA	TGCTGATAGG	AGGCAAGATC	GGTACAATTC	ACAGACAGAC	CAGAATTGGG	TCGCTGAACT	TCGCGATTTG	TCCATTCAAG	CTGAGAATGT	ACTAGAAAGA	240
	**********	**********	**********	**********	**********	**********	**********	**********	**********	**********	**********	**********	
Reference	TATGTCATTG	AAGTGGTGTC	CAGAAGAGAA	GGAAAAGGCC	TTAAAAGGGT	TCTCAAAAGA	TTCACTTGTA	TATTGAACGA	GTGGTCGAGA	ATGCACCAAA	TCGGAGAAGA	CATTAAAGAT	360
Goenbaek	TATGTCATTG	AAGTGGTGTC	CAGAAGAGAA	GGAAAAGGCC	TTAAAAGGGT	TCTCAAAAGA	TTCACTTGTA	TATTGAACGA	GTGGTCGAGA	ATGCACCAAA	TCGGAGAAGA	CATTAAAGAT	360
Osan	TATGTCATTG	AAGTGGTGTC	CAGAAGAGAA	GGAAAAGGCC	TTAAAAGGGT	TCTCAAAAGA	TTCACTTGTA	TATTGAACGA	GTGGTCGAGA	ATGCACCAAA	TCGGAGAAGA	CATTAAAGAT	360
	**********	**********	**********	**********	**********	**********	**********	**********	**********	**********	**********	**********	
Reference	ATCAAGTCTC	GTATGTCCGA	CCTCACCAAA	CAGTCGAAGT	CCATGAGTGT	AGGAGACAAC	TCATCAAGTT	TGGTAGATGA	TATCGATTGG	TCGAGAAGAA	CGTATGGGCA	TGAGGCTGAA	480
Goenbaek	ATCAAGTCTC	GTATGTCCGA	CCTCACCAAA	CAGTCGAAGT	CCATGAGTGT	AGGAGACAAC	TCATCAAGTT	TGGTAGATGA	TATCGATTGG	TCGAGAAGAA	CGTATGGGCA	TGAGGCTGAA	480
Osan	ATCAAGTCTC	GTATGTCCGA	CCTCACCAAA	CAGTCGAAGT	CCATGAGTGT	AGGAGACAAC	TCATCAAGTT	TGGTAGATGA	TATCGATTGG	TCGAGAAGAA	CGTATGGGCA	TGAGGCTGAA	480
	**********	**********	**********	**********	**********	**********	**********	**********	**********	**********	**********	**********	
Reference	AAATATTTTG	TAGGAATGAA	GGAGGATATT	AAACAGTTAG	AATCAGTTAT	GACAACTGAT	GACAAATCAA	ATGGAGTGAT	TTCCATATGT	GGCATGGGAG	GCTTGGGAAA	GACCACTCTT	600
Goenbaek	AAATATTTTG	TAGGAATGAA	GGAGGATATT	AAACAGTTAG	AATCAGTTAT	GACAACTGAT	GACAAATCAA	ATGGAGTGAT	TTCCATATGT	GGCATGGGAG	GCTTGGGAAA	GACCACTCTT	600
Osan	AAATATTTTG	TAGGAATGAA	GGAGGATATT	AAACAGTTAG	AATCAGTTAT	GACAACTGAT	GACAAATCAA	ATGGAGTGAT	TTCCATATGT	GGCATGGGAG	GCTTGGGAAA	GACCACTCTT	600
	**********	**********	**********	**********	**********	**********	**********	**********	**********	**********	**********	**********	
Reference	GCTACTAAAA	TTTACAATGG	GGAGGCCGTG	CAGCGATGCT	TCAAATATCG	TGCTTGGGTT	TGTGTAAGCC	AGCAATTTCA	ACCTAGAACT	ATTTTCCAAT	GACTATTAAA	GCAACTTCTT	720
Goenbaek	GCTACTAAAA	TTTACAATGG	GGAGGCCGTG	CAGCGATGCT	TCAAATATCG	TGCTTGGGTT	TGTGTAAGCC	AGCAATTTCA	ACCTAGAACT	ATTTTCCAAT	GACTATTAAA	GCAACTTCTT	720
Osan	GCTACTAAAA	TTTACAATGG	GGAGGCCGTG	CAGCGATGCT	TCAAATATCG	TGCTTGGGTT	TGTGTAAGCC	AGCAATTTCA	ACCTAGAACT	ATTTTCCAAT	GACTATTAAA	GCAACTTCTT	720
	**********	**********	**********	**********	**********	**********	**********	**********	**********	**********	**********	**********	
Reference	CCAAATGAAA	CAGAGGAGCA	AGACGAAGAT	GCATTGGTCA	GAAAGTTGTA	TGATGTACAA	AAAAATAGAA	AATGTCTAAT	AGTTTTGGAT	GACGTTTGGG	AAGTTGATCA	CTGGAATTCC	840
Goenbaek	CCAAATGAAA	CAGAGGAGCA	AGACGAAGAT	GCATTGGTCA	GAAAGTTGTA	TGATGTACAA	AAAAATAGAA	AATGTCTAAT	AGTTTTGGAT	GACGTTTGGG	AAGTTGATCA	CTGGAATTCC	840
Osan	CCAAATGAAA	CAGAGGAGCA	AGACGAAGAT	GCATTGGTCA	GAAAGTTGTA	TGATGTACAA	AAAAATAGAA	AATGTCTAAT	AGTTTTGGAT	GACGTTTGGG	AAGTTGATCA	CTGGAATTCC	840
	**********	**********	**********	**********	**********	**********	**********	**********	**********	**********	**********	**********	

```
Reference  TTAAGGCATG  CATTTCCCAT  TGGAGAGGCA  GATAGCAAAG  TATTGCTCAC  AACCAGAAAT  CAAAACATTG  CTTCCACAGG  ATACGTCCAC  AATCTGAAGT  GTTTGGATGA  AGATGAATGA  960
Goenbaek   TTAAGGCATG  CATTTCCCAT  TGGAGAGGCA  GATAGCAAAG  TATTGCTCAC  AACCAGAAAT  CAAAACATTG  CTTCCACAGG  ATACGTCCAC  AATCTGAAGT  GTTTGGATGA  AGATGAATGA  960
Osan       TTAAGGCATG  CATTTCCCAT  TGGAGAGGCA  GATAGCAAAG  TATTGCTCAC  AACCAGAAAT  CAAAACATTG  CTTCCACAGG  ATACGTCCAC  AATCTGAAGT  GTTTGGATGA  AGATGAATGA  960
           **********  **********  **********  **********  **********  **********  **********  **********  **********  **********  **********  **********

Reference  TGGGAGCTCC  TTCAAAAGAT  AGCACTCCCA  AACAACTATT  CACAAGGTTT  GTTTGTAAAA  TTTCTTGTTT  TATTTTATTG  ATACTAAAAA  TATCATAGTA  TATATTAAGC  GAAAAAGATT  1080
Goenbaek   TGGGAGCTCC  TTCAAAAGAT  AGCACTCCCA  AACAACTATT  CACAAGGTTT  GTTTGTAAAA  TTTCTTGTTT  TATTTTATTG  ATACTAAAAA  TATCATAGTA  TATATTAAGC  GAAAAAGATT  1080
Osan       TGGGAGCTCC  TTCAAAAGAT  AGCACTCCCA  AACAACTATT  CACAAGGTTT  GTTTGTAAAA  TTTCTTGTTT  TATTTTATTG  ATACTAAAAA  TATCATAGTA  TATATTAAGC  GAAAAAGATT  1080
           **********  **********  **********  **********  **********  **********  **********  **********  **********  **********  **********  **********

Reference  ATTTTGTTAC  GTCTAATATT  TTTTTATAGT  TTCCACGACG  ACAAGATTGT  TTGAATTTTT  GCTTATAAAA  AGATTGTTTG  AATTTTCTTT  TGTAAATTTT  AAGTTGATCT  ATATGTATAA  1200
Goenbaek   ATTTTGTTAC  GTCTAATATT  TTTTTATAGT  TTCCACGACG  ACAAGATTGT  TTGAATTTTT  GCTTATAAAA  AGATTGTTTG  AATTTTCTTT  TGTAAATTTT  AAGTTGATCT  ATATGTATAA  1200
Osan       ATTTTGTTAC  GTCTAATATT  TTTTTATAGT  TTCCACGACG  ACAAGATTGT  TTGAATTTTT  GCTTATAAAA  AGATTGTTTG  AATTTTCTTT  TGTAAATTTT  AAGTTGATCT  ATATGTATAA  1200
           **********  **********  **********  **********  **********  **********  **********  **********  **********  **********  **********  **********

Reference  AATGCTATTG  AATAGCAATT  TAAAATTTAT  TACTTCATAC  AAAATTTATT  TCCAACATAA  AAGAAATCAA  ACATTTAGAA  ACTCATTGAA  TTTGTTTCAT  ACAGTAATAT  AAAGTTATGC  1320
Goenbaek   AATGCTATTG  AATAGCAATT  TAAAATTTAT  TACTTCATAC  AAAATTTATT  TCCAACATAA  AAGAAATCAA  ACATTTAGAA  ACTCATTGAA  TTTGTTTCAT  ACAGTAATAT  AAAGTTATGC  1320
Osan       AATGCTATTG  AATAGCAATT  TAAAATTTAT  TACTTCATAC  AAAATTTATT  TCCAACATAA  AAGAAATCAA  ACATTTAGAA  ACTCATTGAA  TTTGTTTCAT  ACAGTAATAT  AAAGTTATGC  1320
           **********  **********  **********  **********  **********  **********  **********  **********  **********  **********  **********  **********

Reference  TTTCAGATTT  TATTTCTATT  TTTTATTTAT  TGCAAGTATA  GGGCAAAATT  CTCTTGTTTT  GATTTGTTTT  AATTACTATA  CAAATTTATA  AGAAATTCAA  ATTTTATAAT  ATATTGAATT  1440
Goenbaek   TTTCAGATTT  TATTTCTATT  TTTTATTTAT  TGCAAGTATA  GGGCAAAATT  CTCTTGTTTT  GATTTGTTTT  AATTACTATA  CAAATTTATA  AGAAATTCAA  ATTTTATAAT  ATATTGAATT  1440
Osan       TTTCAGATTT  TATTTCTATT  TTTTATTTAT  TGCAAGTATA  GGGCAAAATT  CTCTTGTTTT  GATTTGTTTT  AATTACTATA  CAAATTTATA  AGAAATTCAA  ATTTTATAAT  ATATTGAATT  1440
           **********  **********  **********  **********  **********  **********  **********  **********  **********  **********  **********  **********

Reference  AACTGACATT  CATTGATTGT  GTATGCAGAG  CTACCTACAA  CTGAAATAAA  GTTGTTGGAA  GAATATGGAA  GGGAAATAGT  AAAAAAATGT  GGTTATTTAC  CATTACCCAT  CTCAGTTATT  1560
Goenbaek   AACTGACATT  CATTGATTGT  GTATGCAGAG  CTACCTACAA  CTGAAATAAA  GTTGTTGGAA  GAATATGGAA  GGGAAATAGT  AAAAAAATGT  GGTTATTTAC  CATTACCCAT  CTCAGTTATT  1560
Osan       AACTGACATT  CATTGATTGT  GTATGCAGAG  CTACCTACAA  CTGAAATAAA  GTTGTTGGAA  GAATATGGAA  GGGAAATAGT  AAAAAAATGT  GGTTATTTAC  CATTACCCAT  CTCAGTTATT  1560
           **********  **********  **********  **********  **********  **********  **********  **********  **********  **********  **********  **********

Reference  GGGGGAACTC  TTCGTCGTGA  AAAGGCATAA  TTAGAATGGA  AAAATGTGTG  TAGAAATCTT  GATTCGTACC  TCCAACATGG  GAAAGGTTTG  GAGAATGACA  AAAAAGTAAA  TCAAATACTA  1680
Goenbaek   GGGGGAACTC  TTCGTCGTGA  AAAGGCATAA  TTAGAATGGA  AAAATGTGTG  TAGAAATCTT  GATTCGTACC  TCCAACATGG  GAAAGGTTTG  GAGAATGACA  AAAAAGTAAA  TCAAATACTA  1680
```

Osan	GGGGGAACTC	TTCGTCGTGA	AAAGGCATAA	TTAGAATGGA	AAAATGTGTG	TAGAAATCTT	GATTCGTACC	TCCAACATGG	GAAAGGTTTG	GAGAATGACA	AAAAAGTAAA	TCAAATACTA	1680
	**********	**********	**********	**********	**********	**********	**********	**********	**********	**********	**********	**********	
Reference	GATTTGAGTT	ACAATGTGCT	CCCTTACAAC	CTGAAACCAT	GCTTTTTGTA	TTTGGCATGT	TTTAAAGAGG	ATCAAGAAAT	AGATACAGAA	AAACTATATT	TACTATGGAT	GGCTGAAGGA	1800
Goenbaek	GATTTGAGTT	ACAATGTGCT	CCCTTACAAC	CTGAAACCAT	GCTTTTTGTA	TTTGGCATGT	TTTAAAGAGG	ATCAAGAAAT	AGATACAGAA	AAACTATATT	TACTATGGAT	GGCTGAAGGA	1800
Osan	GATTTGAGTT	ACAATGTGCT	CCCTTACAAC	CTGAAACCAT	GCTTTTTGTA	TTTGGCATGT	TTTAAAGAGG	ATCAAGAAAT	AGATACAGAA	AAACTATATT	TACTATGGAT	GGCTGAAGGA	1800
	**********	**********	**********	**********	**********	**********	**********	**********	**********	**********	**********	**********	
Reference	ATGATTTCTT	CAGAAGATAA	GGGAAGGGGA	GAAAGTTTGA	GAGATGTGGG	AGAACGGTAT	TTATTCGAGC	TAGCAAATAG	GTGTTTGGTT	CAAGTGGAAA	TAGACGAGTT	GCCGCTTTAT	1920
Goenbaek	ATGATTTCTT	CAGAAGATAA	GGGAAGGGGA	GAAAGTTTGA	GAGATGTGGG	AGAACGGTAT	TTATTCGAGC	TAGCAAATAG	GTGTTTGGTT	CAAGTGGAAA	TAGACGAGTT	GCCGCTTTAT	1920
Osan	ATGATTTCTT	CAGAAGATAA	GGGAAGGGGA	GAAAGTTTGA	GAGATGTGGG	AGAACGGTAT	TTATTCGAGC	TAGCAAATAG	GTGTTTGGTT	CAAGTGGAAA	TAGACGAGTT	GCCGCTTTAT	1920
	**********	**********	**********	**********	**********	**********	**********	**********	**********	**********	**********	**********	
Reference	AATAGGTTTA	AGTCATGTCG	GCTTCATGAT	TTGATTAGAG	ATCTATGTTT	GTCAAAAGGG	AAAAGACAAG	GATTTTTGGA	GGTTATGGAT	AGAGAGATGG	GAGGAGGAGA	CTCTTCCATT	2040
Goenbaek	AATAGGTTTA	AGTCATGTCG	GCTTCATGAT	TTGATTAGAG	ATCTATGTTT	GTCAAAAGGG	AAAAGACAAG	GATTTTTGGA	GGTTATGGAT	AGAGAGATGG	GAGGAGGAGA	CTCTTCCATT	2040
Osan	AATAGGTTTA	AGTCATGTCG	GCTTCATGAT	TTGATTAGAG	ATCTATGTTT	GTCAAAAGGG	AAAAGACAAG	GATTTTTGGA	GGTTATGGAT	AGAGAGATGG	GAGGAGGAGA	CTCTTCCATT	2040
	**********	**********	**********	**********	**********	**********	**********	**********	**********	**********	**********	**********	
Reference	TGCAAAACAG	ATAGACTGGC	TATCTATATG	CACAGATTGG	ATAATGATCT	TAGTTATAGG	ATTGGAGAAA	ATAAGAACAT	AAGATCTCTT	CTATTCCTCA	AAACGGACTG	GGGAAATATA	2160
Goenbaek	TGCAAAACAG	ATAGACTGGC	TATCTATATG	CACAGATTGG	ATAATGATCT	TAGTTATAGG	ATTGGAGAAA	ATAAGAACAT	AAGATCTCTT	CTATTCCTCA	AAACGGACTG	GGGAAATATA	2160
Osan	TGCAAAACAG	ATAGACTGGC	TATCTATATG	CACAGATTGG	ATAATGATCT	TAGTTATAGG	ATTGGAGAAA	ATAAGAACAT	AAGATCTCTT	CTATTCCTCA	AAACGGACTG	GGGAAATATA	2160
	**********	**********	**********	**********	**********	**********	**********	**********	**********	**********	**********	**********	
Reference	GTTTGGTACA	ATTACTTTAC	ATTTGGGATT	TTCAAATCTC	TCAAAGTTTT	AGTATTGGAA	GGTTATTCAT	TTGAGAATCT	GAAATTGCCC	AAAGGAATAG	AAAAATTGAA	GCTGTTGAAG	2280
Goenbaek	GTTTGGTACA	ATTACTTTAC	ATTTGGGATT	TTCAAATCTC	TCAAAGTTTT	AGTATTGGAA	GGTTATTCAT	TTGAGAATCT	GAAATTGCCC	AAAGGAATAG	AAAAATTGAA	GCTGTTGAAG	2280
Osan	GTTTGGTACA	ATTACTTTAC	ATTTGGGATT	TTCAAATCTC	TCAAAGTTTT	AGTATTGGAA	GGTTATTCAT	TTGAGAATCT	GAAATTGCCC	AAAGGAATAG	AAAAATTGAA	GCTGTTGAAG	2280
	**********	**********	**********	**********	**********	**********	**********	**********	**********	**********	**********	**********	
Reference	CTATTGAGTA	TCGAAAATAG	TACTGTGAAA	GAATTGCCAG	CATCTATATG	CAAGCTACCT	TGTTTGCAGA	TATTGAATGT	GAAACATACA	TTTAGATTAC	CTAATTGCGT	ATACAAAATG	2400
Goenbaek	CTATTGAGTA	TCGAAAATAG	TACTGTGAAA	GAATTGCCAG	CATCTATATG	CAAGCTACCT	TGTTTGCAGA	TATTGAATGT	GAAACATACA	TTTAGATTAC	CTAATTGCGT	ATACAAAATG	2400
Osan	CTATTGAGTA	TCGAAAATAG	TACTGTGAAA	GAATTGCCAG	CATCTATATG	CAAGCTACCT	TGTTTGCAGA	CATTAAATGT	GAAACATACA	TTTAGATTAC	CTAATTGCGT	ATACAAAATG	2400
	**********	**********	**********	**********	**********	**********	**********	:***:*****	**********	**********	**********	**********	
Reference	AGGCGCTTGA	GGCATCTATT	TTTGCACCAT	GATCATAAGA	GCATTGGAGG	TGAAAAATTG	AAATTGGAAG	GGTTGAATGA	GTTGGAGATG	ATAACTGGGT	TCAAGAGTTT	GGTTGATGAC	2520

Goenbaek	AGGCGCTTGA	GGCATCTATT	TTTGCACCAT	GATCATAAGA	GCATTGGAGG	TGAAAAATTG	AAATTGGAAG	GGTTGAATGA	GTTGGAGATG	ATAACTGGGT	TCAAGAGTTT	GGTTGATGAC	2520
Osan	AGGCGCTTGA	GGCATCTATT	TTTGCACCAT	GATCATAAGA	GCATTGGAGG	TGAAAAATTG	AAATTGGAAG	GGTTGAATGA	GTTGGAGATG	ATAACTGGGT	TCAAGAGTTT	GGTTGATGAC	2520
	**********	**********	**********	**********	**********	**********	**********	**********	**********	**********	**********	**********	
Reference	ATCACTCATC	TTCTTAAATT	GCCGAAACTC	CGAGTATTGG	AAGGAAGAAT	TTGTGATGAA	GAAAGTTTGT	CAATGATTGT	TGATCACATC	TTAAATCATC	AAGAACAATT	TCGCGACGTA	2640
Goenbaek	ATCACTCATC	TTCTTAAATT	GCCGAAACTC	CGAGTATTGG	AAGGAAGAAT	TTGTGATGAA	GAAAGTTTGT	CAATGATTGT	TGATCACATC	TTAAATCATC	AAGAACAATT	TCGCGACGTA	2640
Osan	ATCACTCATC	TTCTTAAATT	GCCGAAACTC	CGAGTATTGG	AAGGAAGAAT	TTGTGATGAA	GAAAGTTTGT	CAATGATTGT	TGATCACATC	TTAAATCATC	AAGAACAATT	TCGCGACGTA	2640
	**********	**********	**********	**********	**********	**********	**********	**********	**********	**********	**********	**********	
Reference	CGACTTCAGA	TTGAAATGGA	TGTTAATATG	GATTCGGAAG	ATGGCTCAAC	TCTTCTCAAA	AGGTTGGTGA	CGTGTCACTC	GCTACATTAC	TTGAGAATCG	CACATTGTCA	AGTGAGCAAA	2760
Goenbaek	CGACTTCAGA	TTGAAATGGA	TGTTAATATG	GATTCGGAAG	ATGGCTCAAC	TCTTCTCAAA	AGGTTGGTGA	CGTGTCACTC	GCTACATTAC	TTGAGAATCG	CACATTGTCA	AGTGAGCAAA	2760
Osan	CGACTTCAGA	TTGAAATGGA	TGTTAATATG	GATTCGGAAG	ATGGCTCAAC	TCTTCTCAAA	AGGTTGGTGA	CGTGTCACTC	GCTACATTAC	TTGAGAATCG	CACATTGTCA	AGTGAGCAAA	2760
	**********	**********	**********	**********	**********	**********	**********	**********	**********	**********	**********	**********	
Reference	TTACCAGCTT	ATGAAGTTCA	ACTATATCAA	AATGTGATAG	AATTGCACCT	TGTGGGTACA	AGGATTGAGG	AAGACCCAAT	GGAAATACTA	GAGAAGCTTC	CCATGTTAAG	AGTTCTTGGC	2880
Goenbaek	TTACCAGCTT	ATGAAGTTCA	ACTATATCAA	AATGTGATAG	AATTGCACCT	TGTGGGTACA	AGGATTGAGG	AAGACCCAAT	GGAAATACTA	GAGAAGCTTC	CCATGTTAAG	AGTTCTTGGC	2880
Osan	TTACCAGCTT	ATGAAGTTCA	ACTATATCAA	AATGTGATAG	AATTGCACCT	TGTGGGTACA	AGGATTGAGG	AAGACCCAAT	GGAAATACTA	GAGAAGCTTC	CCATGTTAAG	AGTTCTTGGC	2880
	**********	**********	**********	**********	**********	**********	**********	**********	**********	**********	**********	**********	
Reference	TTGTGGCGCA	ATCCATATAT	GGGCAATGAG	ATGGTTTGTC	GGGCAACTGG	ATTTCCTCAA	CTCAGAGACC	TTGTTCTGTT	TGGGTTGTCG	AATTTAGTGG	AATGGAGAGT	GGAGAAAGGA	3000
Goenbaek	TTGTGGCGCA	ATCCATATAT	GGGCAATGAG	ATGGTTTGTC	GGGCAACTGG	ATTTCCTCAA	CTCAGAGACC	TTGTTCTGTT	TGGGTTGTCG	AATTTAGTGG	AATGGAGAGT	GGAGAAAGGA	3000
Osan	TTGTGGCGCA	ATCCATATAT	GGGCAATGAG	ATGGTTTGTC	GGGCAACTGG	ATTTCCTCAA	CTCAGAGACC	TTGTTCTGTT	TGGGTTGTCG	AATTTAGTGG	AATGGAGAGT	GGAGAAAGGA	3000
	**********	**********	**********	**********	**********	**********	**********	**********	**********	**********	**********	**********	
Reference	GCAATGCTCA	ATCTATCTTT	TCTTTATATT	AAAGCATGCA	GAAAATTGGC	GATGATTCCA	GATGGATTGA	AATTCATCTC	TACTCTCAAA	GAAATGGAAA	TTATGTTAAT	GCCACAAGAG	3090
Goenbaek	GCAATGCTCA	ATCTATCTTT	TCTTTATATT	AAAGCATGCA	GAAAATTGGC	GATGATTCCA	GATGGATTGA	AATTCATCTC	TACTCTCAAA	GAAATGGAAA	TTATGTTAAT	GCCACAAGAG	3090
Osan	GCAATGCTCA	ATCTATCTTT	TCTTTATATT	AAAGCATGCA	GAAAATTGGC	GATGATTCCA	GATGGATTGA	AATTCATCTC	TACTCTCAAA	GAAATGGAAA	TTATGTTAAT	GCCACAAGAG	3090
	**********	**********	**********	**********	**********	**********	**********	**********	**********	**********	**********	**********	
Reference	TTCATGAAGA	GGGTACAAGT	GGTGGATGGT	GAAGAAGGAG	AAGATTATCA	CAAAATCAAA	CACATACCTT	TCATTTACAT	TTATAATTAA				3210
Goenbaek	TTCATGAAGA	GGGTACAAGT	GGTGGATGGT	GAAGAAGGAG	AAGATTATCA	CAAAATCAAA	CACATACCTT	TCATTTACAT	TTATAATTAA				3210
Osan	TTCATGAAGA	GGGTACAAGT	GGTGGATGGT	GAAGAAGGAG	AAGATTATCA	CAAAATCAAA	CACATACCTT	TCATTTACAT	TTATAATTAA				3210
	**********	**********	**********	**********	**********	**********	**********	**********	**********				

SUPPLEMENTARY FIGURE S6. Single nucleotide polymorphism (SNPs) among Goenbaek, Osan, and sesame reference genome (Zhongzhi13) sequence of *SIN_1019016.*

SUPPLEMENTARY TABLE S1. Details of the germplasm lines used in the association analysis in the present study.

Accession	Breeding status	Country of Origin	KACC48121		KACC48120		No2526		Reference
			Disease[a] score	Resistance[b] level	Disease score	Resistance level	Disease score	Resistance level	
Dodam	Cultivar	Korea	0.0	R	0.0	R	0.0	R	Oh et al., 2018
Geonbaek	Cultivar	Korea	0.0	R	0.0	R	0.9	R	Oh et al., 2018
Superteagang	Commercial cultivar	Korea	0.0	R	0.0	R	-	-	present study
Jinju	Cultivar	Korea	0.0	R	0.0	R	0.3	R	Oh et al., 2018
SIG960320-1A	Breeding line	Korea	5.0	MR	2.7	R	2.0	R	Oh et al., 2018
Gochang collection	Landrace	Korea	0.0	R	0.0	R	-	-	present study
Jinmi	Cultivar	Korea	0.0	R	9.0	S	-	-	present study
Chamhwang	Cultivar	Korea	0.0	R	0.0	R	4.5	MR	Oh et al., 2018
Wild sesame	*S. alatum*/wild	Tropical Africa, India	0.0	R	0.0	R	0.0	R	present study
Naman	Cultivar	Korea	0.1	R	0.0	R	0.1	R	Oh et al., 2018
Pungan	Cultivar	Korea	0.4	R	0.1	R	0.2	R	Oh et al., 2018
Namsan	Cultivar	Korea	0.8	R	0.3	R	0.3	R	Oh et al., 2018
Jungmo 5002	Cultivar	Korea	0.9	R	0.1	R	0.1	R	Oh et al., 2018
Jinbaek	Cultivar	Korea	1.4	R	0.0	R	0.0	R	Oh et al., 2018
HS445-1-1-2-2-4	Breeding line	Korea	1.5	R	9.0	S	9.0	S	Oh et al., 2018
Yoomi	Cultivar	Korea	1.6	R	0.5	R	0.0	R	Oh et al., 2018
Konheuk	Cultivar	Korea	1.2	R	5.2	MS	8.0	S	Oh et al., 2018
Sungbun	Cultivar	Korea	1.8	R	8.2	S	5.7	MS	Oh et al., 2018
Gangheuk	Cultivar	Korea	1.8	R	0.2	R	0.5	R	Oh et al., 2018
Suwon	Cultivar	Korea	2.6	R	8.5	S	8.8	S	Oh et al., 2018
Hansan	Cultivar	Korea	3.0	R	8.8	S	9.0	S	Oh et al., 2018
Pungyeon	Cultivar	Korea	4.0	MR	8.2	S	9.0	S	Oh et al., 2018
NonggiS-4	Landrace	Japan	4.3	MR	4.3	MR	3.0	R	Oh et al., 2018

cont.

Accession	Breeding status	Country of Origin	KACC48121		KACC48120		No2526		Reference
			Disease score	Resistance level	Disease score	Resistance level	Disease score	Resistance level	
Namda	Cultivar	Korea	4.8	MR	8.5	S	8.2	S	Oh et al., 2018
IT184749	Landrace	United States	5.0	MR	6.8	MS	-	-	present study
Pungsan	Cultivar	Korea	5.2	MS	8.6	S	9.0	S	Oh et al., 2018
Yuseong	Cultivar	Korea	5.3	MS	8.2	S	8.0	S	Oh et al., 2018
Seodun	Cultivar	Korea	5.8	MS	8.8	S	7.6	S	Oh et al., 2018
Ahnsan	Cultivar	Korea	5.8	MS	8.7	S	9.0	S	Oh et al., 2018
Yehan	Cultivar	Korea	6.0	MS	6.1	MS	8.6	S	Oh et al., 2018
Heuksun	Cultivar	Korea	6.2	MS	8.6	S	7.2	S	Oh et al., 2018
Nambaek	Cultivar	Korea	6.2	MS	8.6	S	9.0	S	Oh et al., 2018
Hanseom	Cultivar	Korea	6.2	MS	7.8	S	9.0	S	Oh et al., 2018
Suji	Cultivar	Korea	6.3	MS	7.5	MS	9.0	S	Oh et al., 2018
Jinyul	Cultivar	Korea	6.5	MS	6.3	MS	9.0	S	Oh et al., 2018
Danbaek	Cultivar	Korea	6.6	MS	8.1	S	9.0	S	Oh et al., 2018
dt-45	mutant	Israel	7.2	MS	9.0	S	9.0	MS	Oh et al., 2018
Pungsung	Cultivar	Korea	7.4	S	7.1	S	9.0	S	Oh et al., 2018
Yupung	Cultivar	Korea	7.4	S	0.1	R	9.0	S	Oh et al., 2018
Manli	Cultivar	Korea	8.0	S	8.1	S	8.8	S	Oh et al., 2018
Mangeum	Cultivar	Korea	7.8	S	7.3	S	9.0	S	Oh et al., 2018
Yangan	Cultivar	Korea	7.8	S	6.8	MS	9.0	S	Oh et al., 2018
Jungmo 5003	Cultivar	Korea	8.0	S	0.3	R	9.0	S	Oh et al., 2018
Manhuk	Cultivar	Korea	7.8	S	8.7	S	6.4	MS	Oh et al., 2018

Hwaryong	Cultivar	Korea	8.0	S	6.7	MS	9.0	S	Oh et al., 2018

cont.

Accession	Breeding status	Country of Origin	KACC48121		KACC48120		No2526		Reference
			Disease score	Resistance level	Disease score	Resistance level	Disease score	Resistance level	
Dubeol	Cultivar	Korea	8.1	S	5.7	MS	8.8	S	Oh et al., 2018
Baeksun	Cultivar	Korea	9.0	S	6.4	MS	9.0	S	Oh et al., 2018
Jungmo 5007	Cultivar	Korea	8.8	S	8.5	S	9.0	S	Oh et al., 2018
Kangan	Cultivar	Korea	8.6	S	7.1	MS	9.0	S	Oh et al., 2018
Yubaek	Cultivar	Korea	8.6	S	7.4	S	9.0	S	Oh et al., 2018
Sangbaek	Cultivar	Korea	8.6	S	0.0	R	9.0	S	Oh et al., 2018
Kwangsan	Cultivar	Korea	7.8	S	8.6	S	9.0	S	Oh et al., 2018
Milsung	Cultivar	Korea	8.6	S	7.4	S	9.0	S	Oh et al., 2018
Galmi	Cultivar	Korea	8.6	S	9.0	S	9.0	S	Oh et al., 2018
Daheuk	Cultivar	Korea	8.8	S	9.0	S	9.0	S	Oh et al., 2018
Sunheuk	Cultivar	Korea	7.6	S	4.0	MR	9.0	S	Oh et al., 2018
Hwangbaek	Cultivar	Korea	8.6	S	8.9	S	9.0	S	Oh et al., 2018
Pungnam	Cultivar	Korea	8.7	S	8.8	S	9.0	S	Oh et al., 2018
Hogeon	Cultivar	Korea	8.8	S	8.1	S	9.0	S	Oh et al., 2018
Ahnbaek	Cultivar	Korea	8.8	S	8.3	S	8.8	S	Oh et al., 2018
Yangheuk	Cultivar	Korea	8.8	S	8.5	S	9.0	S	Oh et al., 2018
PI 157156	Landrace	India	9.0	S	-	-	-	-	present study
dt-sel	mutant	Israel	9.0	S	9.0	S	9.0	S	Oh et al., 2018
Gomazou	Cultivar	Japan	9.0	S	9.0	S	9.0	S	present study
Kyeonbuk 27	Breeding line	Korea	9.0	S	-	-	-	-	present study
Olleh	Commercial cultivar	Korea	9.0	S	9.0	S	-	-	present study

Miho	Cultivar	Korea	9.0	S	9.0	S	-	-	present study

cont.

Accession	Breeding status	Country of Origin	KACC48121		KACC48120		No2526		Reference
			Disease score	Resistance level	Disease score	Resistance level	Disease score	Resistance level	
Maniheuk	Commercial cultivar	Korea	9.0	S	9.0	S	-	-	present study
Superansan	Commercial cultivar	Korea	9.0	S	9.0	S	-	-	present study
Annam	Cultivar	Korea	9.0	S	8.5	S	9.0	S	Oh et al., 2018
Buloheuk	Cultivar	Korea	9.0	S	9.0	S	-	-	present study
Heukjanggun	Commercial cultivar	Korea	9.0	S	9.0	S	-	-	present study
Gopum	Cultivar	Korea	9.0	S	8.7	S	9.0	S	present study
Baengmi	Commercial cultivar	Korea	9.0	S	9.0	S	-	-	present study
PI 490033	Landrace	Korea	9.0	S	-	-	-	-	present study
Plusansan	Commercial cultivar	Korea	9.0	S	9.0	S	-	-	present study
Kangbaek	Cultivar	Korea	9.0	S	0.0	R	9.0	S	Oh et al., 2018
Osan	Cultivar	Korea	9.0	S	8.2	S	9.0	S	Oh et al., 2018
Kyeonbuk 33	Breeding line	Korea	9.0	S	9.0	S	-	-	Oh et al., 2018
Miheuk	Cultivar	Korea	9.0	S	8.5	S	9.0	S	Oh et al., 2018
Yunheuk	Cultivar	Korea	9.0	S	8.9	S	9.0	S	Oh et al., 2018
Hwanggeum	Cultivar	Korea	9.0	S	9.0	S	-	-	Oh et al., 2018
Areum	Cultivar	Korea	9.0	S	8.5	S	9.0	S	Oh et al., 2018
Pyoungan	Cultivar	Korea	9.0	S	8.5	S	9.0	S	Oh et al., 2018
PI 279536	Landrace	Mexico	9.0	S	-	-	-	-	present study
PI 599446	Landrace	United States	7.6	S	9.0	S	9.0	S	Oh et al., 2018
Early Russian	Cultivar	United States, Texas	9.0	S	9.0	S	9.0	S	Oh et al., 2018

[a]According to Oh *et al.* (2018) disease scale Average disease severity: 0, no symptom; 1, slight wilt of leaves; 3, wilt and minor blight on leaves; 5, minor discoloration in stem base; 7, clear discoloration of stem and wilting; 9, damping off and withering.
[b]Resistance level: resistant (average score 0-3); MR: moderate resistance (average score 3.1-5); MS: moderate susceptible (average score 5.1-7); S: susceptible (average score 7.1-9).

SUPPLEMENTARY TABLE S2. Segregation ratio and chi-square analysis in F_2 individuals from the cross of Goenbaek × Osan (G×O), and Goenbaek × Milsung (G×M) inoculated with KACC48121.

**Cross (Generation)**	**Resistant (R)**	**Susceptible (S)**	**Total**	**Segregation ratio**	$\chi^{2‡}$	***P***
Goenbaek/Osan (F_2)	128	333	461	1:3	2.07	0.15
Goenbaek/Milsung (F_2)	92	327	419	1:3	1.88	0.17

‡df=1.0; χ^2(0.05, 1) =3.84

SUPPLEMENTARY TABLE S3. Phenotypic evaluation and segregation of the single sequence repeat (SSR) markers segregation in recombinant inbred line (RIL) populations.

	Phenotype					
**Isolate**	**GO-RILs**			**GM-RILs**		
	**No. of resistant plants**	**No. of susceptible plants**	χ^2	**No. of resistant plants**	**No. of susceptible plants**	χ^2
KACC48121	45	45	0.150	90	98	0.340
KACC48120	47	43	0.178	–	–	–
No2526	47	43	0.178	–	–	–

	Genotype					
**Marker**	**GO-RILs**			**GM-RILs**		
	**RR=R**	**rr=S**	**χ2(1:1)**	**RR=R**	**rr=S**	**χ2(1:1)**
SiSSM84917	46	43	0.100ns	90	94	0.090ns
SiSSM85023	46	43	0.100ns	99	86	0.910ns
SiSSM84958	41	48	0.550ns	72	113	9.090*
SiSSM86906	37	49	1.670ns	107	81	3.600ns

χ2, Chi-square = 3.841 at the 5% level ($P < 0.05$).

SUPPLEMENTARY TABLE S4. Detailed information of the genetic map developed by using genotyping-by-sequencing (GBS)-generated single nucleotide polymorphism (SNP) markers.

Chromosome	Number of markers	Map length (cM)	Average marker distance (cM)
Chr1	179	121.39	5.70
Chr2	110	70.21	5.12
Chr3	271	118.14	3.38
Chr4	58	57.48	7.08
Chr5	60	36.68	4.65
Chr6	245	73.48	2.90
Chr7	186	33.77	2.25
Chr8	92	64.81	5.09
Chr9	186	94.57	5.04
Chr10	106	53.58	5.44
Chr11	76	69.17	4.51
Chr12	79	53.42	3.63
Chr13	14	36.67	6.09
**Total**	**1662**	**883.37**	**4.69**

SUPPLEMENTARY TABLE S5. Details of simple sequence repeat markers used for parental polymorphism survey between parental lines Goenbaek, Osan, and Milsung and two contrasting bulks G × O and G × M.

Marker ID	Source	SSR motif	Size	Primer Forward (5'-3')	Primer Reverse (5'-3')	Start (bp)	End (bp)
SiSSM79649	SisatBase	(AG)19	189	TTGTTTTGGTCGGTGTGTGT	CAAGGTTGATGTGGATGGTG	253057	253094
SiSSM79880	SisatBase	(TA)14(GA)10	108	AGAATTCGCATAGGTGTGGG	CTCGTATTATCCGAAACCGC	702935	702982
SiSSM80459	SisatBase	(GA)17	280	GCCTGGAAACGGTTTTAGATT	TCCAAACCCTAACGAAAGAGA	1869257	1869290
SiSSM80534	SisatBase	(AAT)16	256	TGCAGATCATTAAACCTTGAAAAA	TGGTCCAAATCTTTACGAGATG	2007111	2007158
SiSSM80636	SisatBase	(AG)22	150	GTCAATTCCCTTGTCCGAAA	AAGATTAGATGCGCCCTCAA	2187273	2187316
SiSSM81107	SisatBase	(TA)8(GA)13	238	CCATTGAATTTGATTGAGTCTAAGG	GGCCTTTTCTTCCGTCTAGC	3127756	3127797
1643	PMDBase	(AAT)8	273	AAGGTTCGTCGCGTGAATAC	AAAGAGATCACGTGTCAAGCA	3184346	3184369
SiSSM81406	SisatBase	(AT)15	240	TCATTTCTAATCCAAAGGCTGAA	ACCCATATCCCCTATGGAGC	3717471	3717500
SiSSM81434	SisatBase	(GA)15	267	AGCACCAAATGCCAACTACC	ACCTGCGTGAGGAGAGAAGA	3755082	3755111
2040	PMDBase	(AG)14	272	TTATTTGCCGATAGAGACGAA	AGGTGATTGTTTTCCTGCCA	3955904	3955931
SiSSM81764	SisatBase	(AT)17	240	AATTGAACACAAGCCCAAGG	TGCCAGCAAGAACGATTATG	4370928	4370961
2326	PMDBase	(AG)15	191	TGAGAGAGGGTGATTTTGGG	ACAACCTGACCCAACCACAT	4550913	4550942
SiSSM82104	SisatBase	(TA)11(GA)11	200	GAGGCCAAAGCTGTTTTCAG	ACATTGTGACGAACTGCTGC	5364697	5364740
2705	PMDBase	(AT)18	251	TTGAGTGTGCTTCGTTGGTT	GAGGCAGAAGCTGTTTCACC	5768317	5768352
2907	PMDBase	(GA)7gg(GA)9	270	CAAAATTGCAGAAATTCCCC	TTCTGCCTTTTGTTTTCTGTGA	6566921	6566954
3184	PMDBase	(AAT)10	271	GGAGGGAAAGGAATTTGAGG	GGGCAACAAGAAAATGGAGA	7915210	7915239
3627	PMDBase	(AT)7aataagaaag(GA)9	239	GCATAAAGGGGCTAAATGAAA	CCCCACCTTCCACACTTAAC	9886626	9886667
3722	PMDBase	(AAT)11	181	TTATGTCAAAATTTAGTGATTCCTATG	TCGGGTGCTCGAATTTTAAG	10192859	10192891

cont…

Marker ID	Source	SSR motif	Size	Primer Forward (5'-3')	Primer Reverse (5'-3')	Start (bp)	End (bp)
3829	PMDBase	(AT)14	150	TTGCCTCATCATAAGCACAAA	GAACTGCAGGTTTCAGAGGG	10672647	10672674
4014	PMDBase	(AT)17	227	TGAACCAAAAAGCATATTGATAGAA	AAATGTTCACGACATGCACC	11284786	11284819
SiSSM83576	SisatBase	(ATT)14	261	GTTCGGAAATATGCCCAAGA	AAGAGGAGCGTGAGCATTTC	11439728	11439769
5108	PMDBase	(AT)18	280	GGTTGTGCACTAAGGAGTTGC	CAGCTGCACCAACAAAAGTG	13847192	13847227
SiSSM84895	SisatBase	(AT)12	214	TTCTTGGTTGAACTTTGTCTGG	GAATGAATGGATGCAGCAAG	14513502	14513535
**SiSSM84917‡**	**SisatBase**	**(AG)22**	**253**	**GCACACATACACACACGCAC**	**TGTACCACATTCGCCTTGTC**	**14539434**	**14539477**
SiSSM84922	SisatBase	(TA)17	219	TTTCATGAAGCAGCAGAGGA	CGCGATGCAATTTATAACCC	14570408	14570441
SiSSM84923	SisatBase	(A)12	260	TGTTGATCTCTACCCACAACAAA	GATGACGAGACGAGTTTTTCAA	14576365	14576376
SiSSM84926	SisatBase	(AG)8	241	GCAAAGCAAGAATAGCAGGG	GGAAAGAAGAGCCCCAAAAC	14586922	14586937
SiSSM84927	SisatBase	(A)10	276	ACGCCCGCTCTTAACTACG	TCTTCTTCCCCCTCCTCTTC	14588044	14588053
SiSSM84930	SisatBase	compound	178	AGTGGCGATCCCAATTTTAG	TTTATGGGTCCAAATCGACG	14596288	14596393
SiSSM84933	SisatBase	(TG)7	239	GCTTAGGCAAGACCATGTGC	TTCCAGTGGATTCATAGCCC	14598782	14598795
SiSSM84939	SisatBase	compound	268	GAACCGGTGAAAACTTGTCC	ACGACTCTTCCACCCACAAA	14605708	14605789
**SiSSM84958‡**	**SisatBase**	**(CT)15**	**166**	**AGACCCTTTTAGGCTCTGGC**	**AAGCGCTGGATGGAATAGAA**	**14635504**	**14635533**
SiSSM84987	SisatBase	(AC)7	259	GGGTTTGATCCCATGTTGAC	ATGTCCAAAAATTAACGCCG	14701459	14701472
SiSSM84994	SisatBase	compound	196	AAACAGACGGGAGACGAAGA	GGGGAAAGGGCATAATGAAC	14712805	14712854
**SiSSM84996‡**	**SisatBase**	**compound**	**258**	**GGGAGGGAGACAGGGAAATA**	**ATTCGTGGTGATCGAGCTTT**	**14728083**	**14728148**
**SiSSM85023‡**	**SisatBase**	**(TG)10**	**262**	**GGCATGAGCCCACACATAAT**	**AAGACCGACAGCCGATTTTA**	**14765956**	**14765975**

cont…

Marker ID	Source	SSR motif	Size	Primer Forward (5'-3')	Primer Reverse (5'-3')	Start (bp)	End (bp)
SiSSM85041	SisatBase	(GA)11	251	ACGCCAAAATATTTGCCATA	TTCACCACAGCTCATGTTCC	14812838	14812859
SiSSM85056	SisatBase	(AC)6	224	TTGTTAGGAAATGGATCGGG	TGAGACGAGGGTGAGTGTTG	14856950	14856961
SiSSM85088	SisatBase	(GT)9	262	TCGGTGAATTTTGACTGACG	TCCTAAGCAAACGCAGTCCT	14939936	14939953
SiSSM85090	SisatBase	(CT)9	135	CATGACTTATGTATTGCCTATATGTGG	CGGATTGCAGTTTGTCGTTA	14940947	14940964
SiSSM85094	SisatBase	(T)15(G)12	209	TCCTTATGCCTTTGCACTCA	TCGCATAGGATCAAAATCACC	14950201	14950227
SiSSM85229	SisatBase	(TA)17	240	GAGGCACACAGAGGTCACAA	ACTCCTCTAATCCCCCTCCA	15200463	15200496
SiSSM85249	SisatBase	(AT)19	278	TCTCAATTATCAGAAGAAAGTCATAAA	CAAACCCACCTCATTAACCG	15215174	15215211
**SiSSM85354‡**	**SisatBase**	**(GA)15**	**161**	**TGTTCGCCTATTCTGCACTG**	**ACACACACATACACCCACGC**	**15489851**	**15489880**
**SiSSM85570‡**	**SisatBase**	**(GA)17**	**271**	**CTATCTGGGACGACGGAGAG**	**TTTCTTCAACAGATCCCGCT**	**15990137**	**15990170**
SiSSM85784	SisatBase	(AT)12	255	TGATGGCTTTGCTACTCACG	CTGAATTGCCCGTTGTACCT	16364116	16364139
6390	PMDBase	(AG)17	212	GCTTCAGCCAGTTCTCCAAC	CAACCATATGTCGCTGTGCT	16607555	16607588
SiSSM86178	SisatBase	(T)26	235	AGCTATGCACATTTCCACCC	TGTACGTTCCTCGAGCACAA	17182250	17182275
SiSSM86181	SisatBase	(TA)12	235	TGACAAAAAGTGAACCAAAACAA	CATGACGTGGTTCAATGTCC	17185785	17185808
6689	PMDBase	(AT)9(AG)16	145	TAATGGGCAGAGCTAATGGG	GCAAACATGAAGCCTTTGGT	17212236	17212285
SiSSM86323	SisatBase	(AG)14	261	AGCAAAGCACCACCTCAAGT	CCATCCTCTCGCTCTTCTTG	17444408	17444435
**SiSSM86906‡**	**SisatBase**	**(GA)23**	**221**	**AATCAAGATACGACGCAGGG**	**GTCCATTTCTCCTGGCCATA**	**18816333**	**18816378**
7631	PMDBase	(AT)22	193	AGCACCTCTTGTGGTCCATC	TGCAATGAGTGGATTACCGA	19392348	19392391

‡ Polymorphic markers; bold letters indicate polymorphic markers between the two contrasting bulks and parents, Goenbaek (P1), Osan (P2), Milsung (P2)

SUPPLEMENTARY TABLE S6. Candidate resistance genes from the 0.791 Mb genomic region on chromosome 10 significantly associated with genome-wide association study (GWAS) regions and QTLs for Phytophthora blight (PB) resistance and their gene ontology (GO) descriptions.

Chromosome	Gene ID	Start	End	Direction	GO Description§
chromosome 10	SIN_1019027	14,451,847	14,466,672	-	Serine/threonine protein phosphatase 2A regulatory subunit B"beta
chromosome 10	SIN_1019026	14,469,278	14,478,285	-	LOW QUALITY PROTEIN: ubiquitin carboxyl-terminal hydrolase 2
chromosome 10	SIN_1019025	14,483,080	14,483,415	+	Uncharacterized protein LOC110011297
chromosome 10	SIN_1019024	14,484,966	14,487,029	+	Elongation factor 1-alpha-like / hypothetical protein CDL15_Pgr022686
chromosome 10	SIN_1019023	14,489,808	14,493,423	+	Putative disease resistance protein At1g59780
chromosome 10	SIN_1019022	14,509,311	14,512,398	+	Disease resistance protein RPP8-like
chromosome 10	SIN_1019021	14,520,679	14,525,469	+	Small ubiquitin-related modifier 2-like
chromosome 10	SIN_1019020	14,528,970	14,531,008	+	Small ubiquitin-related modifier 1
chromosome 10	SIN_1019019	14,532,148	14,533,208	+	Cytochrome P450 71D95-like / hypothetical protein MIMGU_mgv1a025918mg
chromosome 10	SIN_1019018	14,534,978	14,535,630	+	Small ubiquitin-related modifier 2
chromosome 10	SIN_1019017	14,538,379	14,542,560	-	ER lumen protein-retaining receptor
chromosome 10	SIN_1019016	14,554,076	14,557,285	-	**Probable disease resistance protein At1g58390**
chromosome 10	SIN_1019015	14,560,472	14,564,212	-	Putative disease resistance protein At1g50180
chromosome 10	SIN_1019014	14,570,682	14,570,930	+	Putative F-box protein At1g67623
chromosome 10	SIN_1019013	14,574,206	14,579,023	-	**Proline-rich receptor-like protein kinase PERK3**
chromosome 10	SIN_1019012	14,583,429	14,587,250	-	PLASMODESMATA CALLOSE-BINDING PROTEIN 3
chromosome 10	SIN_1019011	14,601,277	14,601,633	+	Mitogen-activated protein kinase kinase kinase YODA-like
chromosome 10	SIN_1019010	14,603,601	14,604,818	+	Ethylene-responsive transcription factor ERF062
chromosome 10	SIN_1019009	14,609,126	14,609,542	-	Uncharacterized protein
chromosome 10	SIN_1019008	14,630,577	14,634,595	-	High mobility group B protein 15
chromosome 10	SIN_1019007	14,634,704	14,635,027	-	Unknown
chromosome 10	SIN_1019006	14,639,219	14,641,353	-	Repressed By RIM101 protein 1
chromosome 10	SIN_1019005	14,642,509	14,648,243	+	Myosin heavy chain, non-muscle-like
chromosome 10	SIN_1019004	14,650,492	14,654,632	-	Pentatricopeptide repeat-containing protein At4g32430, mitochondrial

chromosome 10	SIN_1019003	14,660,753	14,665,846	+	Uncharacterized protein
chromosome 10	SIN_1019002	14,666,226	14,672,207	-	Uncharacterized protein
chromosome 10	SIN_1019001	14,686,769	14,689,240	+	**Receptor like protein kinase S.2**
chromosome 10	SIN_1019000	14,692,204	14,695,873	-	Protein PHR1-LIKE 2
chromosome 10	SIN_1018999	14,700,937	14,705,072	+	F-box protein PP2-B5
chromosome 10	SIN_1018998	14,706,989	14,711,116	+	Serine/threonine-protein phosphatase PP1
chromosome 10	SIN_1018997	14,724,989	14,727,081	+	F-box protein PP2-B5
chromosome 10	SIN_1018996	14,727,979	14,730,530	+	F-box protein PP2-B5
chromosome 10	SIN_1018995	14,734,025	14,736,558	+	F-box protein PP2-B5
chromosome 10	SIN_1018994	14,737,451	14,746,414	-	Probable pectinesterase 29
chromosome 10	SIN_1018993	14,751,096	14,752,984	-	60S ribosomal protein L13a-4-like
chromosome 10	SIN_1018992	14,766,489	14,767,850	-	Transcription factor FAMA
chromosome 10	SIN_1018991	14,771,856	14,772,131	+	Unknown
chromosome 10	SIN_1018990	14,774,536	14,776,227	-	**Putative disease resistance RPP13-like protein 1**
chromosome 10	SIN_1018989	14,778,561	14,779,476	+	Uncharacterized protein
chromosome 10	SIN_1018988	14,785,577	14,787,307	-	Putative disease resistance protein RGA3
chromosome 10	SIN_1018987	14,789,609	14,790,757	+	Probable disease resistance protein At1g58390
chromosome 10	SIN_1018986	14,793,789	14,796,495	+	Cytochrome b5
chromosome 10	SIN_1018985	14,797,190	14,799,002	-	Cyclin-dependent kinase inhibitor 3
chromosome 10	SIN_1018984	14,803,602	14,805,582	+	Pentatricopeptide Repeat-Containing Protein At4g13650-Like
chromosome 10	SIN_1018983	14,811,413	14,811,823	+	Unknown
chromosome 10	SIN_1018982	14,817,324	14,819,102	+	Unknown
chromosome 10	SIN_1018981	14,820,862	14,821,703	+	Unknown
chromosome 10	SIN_1018980	14,830,448	14,838,296	+	Unnamed protein product
chromosome 10	SIN_1018979	14,839,170	14,845,554	-	Probable disease resistance protein RF45
chromosome 10	SIN_1018978	14,847,198	14,865,712	-	Probable disease resistance protein RF45
chromosome 10	SIN_1018975	14,877,429	14,880,295	+	Cytochrome P450 71A1-like

chromosome 10	SIN_1018974	14,882,704	14,884,595	+	Cytochrome P450 71A1-like
chromosome 10	SIN_1018973	14,888,800	14,893,174	+	Uncharacterized protein
chromosome 10	SIN_1018972	14,903,157	14,904,581	+	Hydroquinone glucosyltransferase-like
chromosome 10	SIN_1018971	14,906,134	14,910,502	+	Cellulose synthase-like protein D1
chromosome 10	SIN_1018970	14,916,358	14,918,488	-	**Vesicle-associated membrane protein 722**
chromosome 10	SIN_1018969	14,920,924	14,923,849	-	Ribonuclease P protein subunit p25-like protein
chromosome 10	SIN_1018968	14,924,429	14,924,668	-	Caffeoylshikimate esterase
chromosome 10	SIN_1018967	14,926,043	14,928,179	+	Uncharacterized aarF domain-containing protein kinase At1g79600
chromosome 10	SIN_1018966	14,929,389	14,935,720	+	Uncharacterized aarF domain-containing protein kinase At1g79600
chromosome 10	SIN_1018965	14,936,297	14,937,565	-	Pentatricopeptide repeat-containing protein At3g26630
chromosome 10	SIN_1018964	14,940,852	14,946,343	+	Ankyrin repeat domain-containing protein 13C-A-like
chromosome 10	SIN_1018963	14,947,161	14,947,986	-	Uncharacterized protein TCM_020382
chromosome 10	SIN_1018962	14,958,873	14,966,719	+	Ubiquinone biosynthesis monooxygenase COQ6
chromosome 10	SIN_1018961	14,971,538	14,973,984	-	Chalcone synthase
chromosome 10	SIN_1018960	14,990,068	14,991,794	-	Chalcone synthase J-like
chromosome 10	SIN_1018959	15,004,626	15,007,378	-	Chalcone synthase-like
chromosome 10	SIN_1018958	15,011,374	15,013,327	+	E3 ubiquitin-protein ligase RLIM
chromosome 10	SIN_1018957	15,027,416	15,032,237	-	3-ketoacyl-CoA thiolase 2, peroxisomal
chromosome 10	SIN_1018956	15,040,719	15,046,302	-	Mitosis inhibitor protein kinase wee1
chromosome 10	SIN_1018955	15,048,690	15,052,668	-	Probable voltage-gated potassium channel subunit beta
chromosome 10	SIN_1018954	15,057,543	15,057,926	+	Uncharacterized protein
chromosome 10	SIN_1018953	15,060,887	15,061,297	+	Uncharacterized protein
chromosome 10	SIN_1018952	15,082,220	15,086,159	+	Probable pectate lyase 8
chromosome 10	SIN_1018951	15,091,397	15,092,469	+	Acanthoscurrin-1
chromosome 10	SIN_1018950	15,094,456	15,094,857	+	Unknown
chromosome 10	SIN_1018949	15,098,454	15,098,759	-	Auxin-induced protein 15A-like
chromosome 10	SIN_1018948	15,102,253	15,102,654	+	Uncharacterized protein

chromosome 10	SIN_1018947	15,103,594	15,104,126	+	Hypothetical protein F511_02833
chromosome 10	SIN_1018946	15,106,078	15,106,702	+	Hypothetical protein F511_02833

§Bold are designed gene-specific primers

SUPPLEMENTARY TABLE S7. List of oligonucleotide primers used for qRT-PCR analysis.

Oligo name	Primer sequence	Product size	Gene name
Phn-10-1-qPCR	F: GGGCACAGAGTTGTTTCCAT<br>R: GATGGCGGTCCAGCTTAATA	210	LOC105171879 receptor like protein kinase S.2
Phn-10-2-qPCR	F: TTTCCCGCTTAACAGGATTG<br>R: AAGTTGCCAAACCATTCAGG	232	LOC105171888 proline-rich receptor-like protein kinase PERK3
Phn-10-3-qPCR	F: CCTGAATGGTTTGGCAACTT<br>R: ACGGGATATGCGAAATCTTG	214	LOC105172053 putative disease resistance RPP13-like protein 1
Phn-10-4-qPCR	F: GCAGAAAATTGGCGATGATT<br>R: ACAATGAATGGAGGGCAAAG	239	**LOC110012696 probable disease resistance protein *At1g58390***
Phn-10-5-qPCR	F: TGAAACTGCCGTAACTAGCATC<br>R:TTCCTCACCTGTGTCCCTTT	175	LOC105171860 vesicle-associated membrane protein 722
*Si*18SrRNA-qPCR	F: CGTCCCTGCCCTTTGTACAC'<br>R: CGAACACTTCACCGGACCAT'		as actin reference gene
